# Effect of human movement on airborne disease transmission in an airplane cabin: study using numerical modeling and quantitative risk analysis

**DOI:** 10.1186/1471-2334-14-434

**Published:** 2014-08-06

**Authors:** Zhuyang Han, Gin Nam Sze To, Sau Chung Fu, Christopher Yu-Hang Chao, Wenguo Weng, Quanyi Huang

**Affiliations:** Institute of Public Safety Research, Department of Engineering Physics, Tsinghua University, Beijing, 100084 China; Department of Mechanical and Aerospace Engineering, The Hong Kong University of Science and Technology, Clear Water Bay, Hong Kong; Building Energy Research Center, Fok Ying Tung Graduate School, The Hong Kong University of Science and Technology, Clear Water Bay, Hong Kong

**Keywords:** Human movement, Aerosol dispersion, Aerodynamic effect, Infectious disease, Risk assessment

## Abstract

**Background:**

Airborne transmission of respiratory infectious disease in indoor environment (e.g. airplane cabin, conference room, hospital, isolated room and inpatient ward) may cause outbreaks of infectious diseases, which may lead to many infection cases and significantly influences on the public health. This issue has received more and more attentions from academics. This work investigates the influence of human movement on the airborne transmission of respiratory infectious diseases in an airplane cabin by using an accurate human model in numerical simulation and comparing the influences of different human movement behaviors on disease transmission.

**Methods:**

The Eulerian–Lagrangian approach is adopted to simulate the dispersion and deposition of the expiratory aerosols. The dose–response model is used to assess the infection risks of the occupants. The likelihood analysis is performed as a hypothesis test on the input parameters and different human movement pattern assumptions. An in-flight SARS outbreak case is used for investigation. A moving person with different moving speeds is simulated to represent the movement behaviors. A digital human model was used to represent the detailed profile of the occupants, which was obtained by scanning a real thermal manikin using the 3D laser scanning system.

**Results:**

The analysis results indicate that human movement can strengthen the downward transport of the aerosols, significantly reduce the overall deposition and removal rate of the suspended aerosols and increase the average infection risk in the cabin. The likelihood estimation result shows that the risk assessment results better fit the outcome of the outbreak case when the movements of the seated passengers are considered. The intake fraction of the moving person is significantly higher than most of the seated passengers.

**Conclusions:**

The infection risk distribution in the airplane cabin highly depends on the movement behaviors of the passengers and the index patient. The walking activities of the crew members and the seated passengers can significantly increase their personal infection risks. Taking the influence of the movement of the seated passengers and the index patient into consideration is necessary and important. For future studies, investigations on the behaviors characteristics of the passengers during flight will be useful and helpful for infection control.

**Electronic supplementary material:**

The online version of this article (doi:10.1186/1471-2334-14-434) contains supplementary material, which is available to authorized users.

## Background

Nowadays, respiratory infectious diseases are threatening the life of humans around the world [1]. Almost 4 million deaths due to respiratory infections diseases and 1.5 million deaths due to tuberculosis are reported every year [2]. In the past four decades, airborne transmission of respiratory infectious diseases within enclosed environment has been widely reported by many epidemiology reports [3–6]. In an airplane cabin, the airflow from front to back of the cabin (longitudinal) is minimal due to the ventilation system, which means in-flight transmission of disease contaminants should be confined within two rows of an infected passenger [6, 7]. One possible cause of the infection of the passengers seated far away from the index patient might be the movements of the walking crew members or passengers along the aisle in the airplane cabin [8]. The walking persons might also get infected when they move close to the infector [5].

Recently, studies on the aerodynamic effects of human movement have received more and more attentions [9]. Significant works have been done on this issue, as summarized in Table 1. In experimental investigations, Matsumoto and Ohba [10], Poussou et al. [11] and Bjørn and Nielsen [12] showed that human movement inside enclosed environments could significantly influence contaminant transport and personal exposures to contaminants. By using Computational Fluid Dynamics (CFD) methods, more studies were performed in different indoor environments. Simple human models [13–15] and realistic walking human models [16, 17] were used as the walking humans in the simulations. Most of these studies [13, 14, 16, 17] used tracer gas as the contaminant, but gaseous contaminants were not accurate enough to represent the transmission of respiration droplets. Wang and Chow [17] and Choi and Edwards [15, 16] studied the influence of human walking on the dispersion and deposition of expiratory droplets in indoor environment. Among previous literatures, significant differences can be found in their methods and conclusions.

**Table 1:** **Previous literatures on the effects of human movement on contaminant transmission**

Author, date	Method	Moving object	Environment	Contaminant	Results
Bjørn and Nielsen [12]	experiment	a life-sized breathing thermal manikin	full-scale test rooms	tracer gas (dinitrogenoxide, N_2_O)	Exhalation and local effects caused by movement may be worth considering if one wishes to contain contaminants in certain areas
Matsumoto and Ohba [10]	experiment	a movable heated object	a full-scale room model	\	The moving object mode and speed showed a significant effect on the air temperature distribution and ventilation effectiveness
Shih et al. [13]	CFD	simple object model	an isolated room	tracer gas (carbon dioxide, CO_2_)	The removal of contaminants was not obviously affected by the moving speed
Choi and Edwards [15]	CFD	a realistic walking human model	a Room–Room and a Room–Hall configuration	particle	The rate of mass transport increases as the walking speed increases, but the total amount of material transported is more influenced by the initial proximity of the human from the doorway.
Mazumdar et al. [14]	CFD	simple object model	a single inpatient ward	tracer gas (sulfur hexafluoride, SF_6_)	The average concentration change in the breathing levels in the ward was generally small
Poussou et al. [11]	experiment	a moving object	a one-tenth scale water-based model	dye	Human movement inside enclosed environments could significantly influence contaminant transport and personal exposures to contaminants.
Mazumdar et al. [8]	CFD	simple object model	an airplane cabin	dye/ tracer gas	The movement of a crew member or a passenger could carry contaminants in its wake to as many rows as the person passed
Wang and Chow [17]	CFD	three different moving human models	an isolation room	expiratory droplets	Human walking disturbed the local velocity field, and the increase of walking speed could effectively reduce the overall number of suspended droplets
Choi and Edwards [16]	CFD	a realistic walking human model	a room compartment	tracer gas (sulfur hexafluoride, SF_6_)	Faster walking speed resulted in less mass transport from the contaminated room into the clean room

According to the transmission mechanism of respiratory infectious disease, the diffusion and dispersion of the aerosols expelled by respiratory activities are important and necessary for infection risk assessment [18]. The airborne transmission of these aerosols in an airplane cabin is significantly different from the diffusion of gaseous contaminants [8, 18, 19]. The methods used for risk assessment of expiratory aerosols and gaseous contaminants are also different [20]. Until now, studies on the effects of human movement on aerosols transmission and infection risk distribution are still rare.

In this work, the effects of human movement on respiratory infectious disease transmission are investigated. An in-flight outbreak case is used for investigation. A manikin with a detailed human profile is also used in the computational geometry. The Eulerian–Lagrangian approach is adopted to simulate the dispersion and deposition of the expiratory aerosols. The infection risks of the occupants are assessed by using quantitative risk analysis, and the influence of human movement on infectious disease transmission is also analyzed. Likelihood analysis is then performed as a hypothesis test on the input parameters and the different human movement pattern assumptions.

## Methods

### The outbreak case and computational geometry

**Figure 1:**
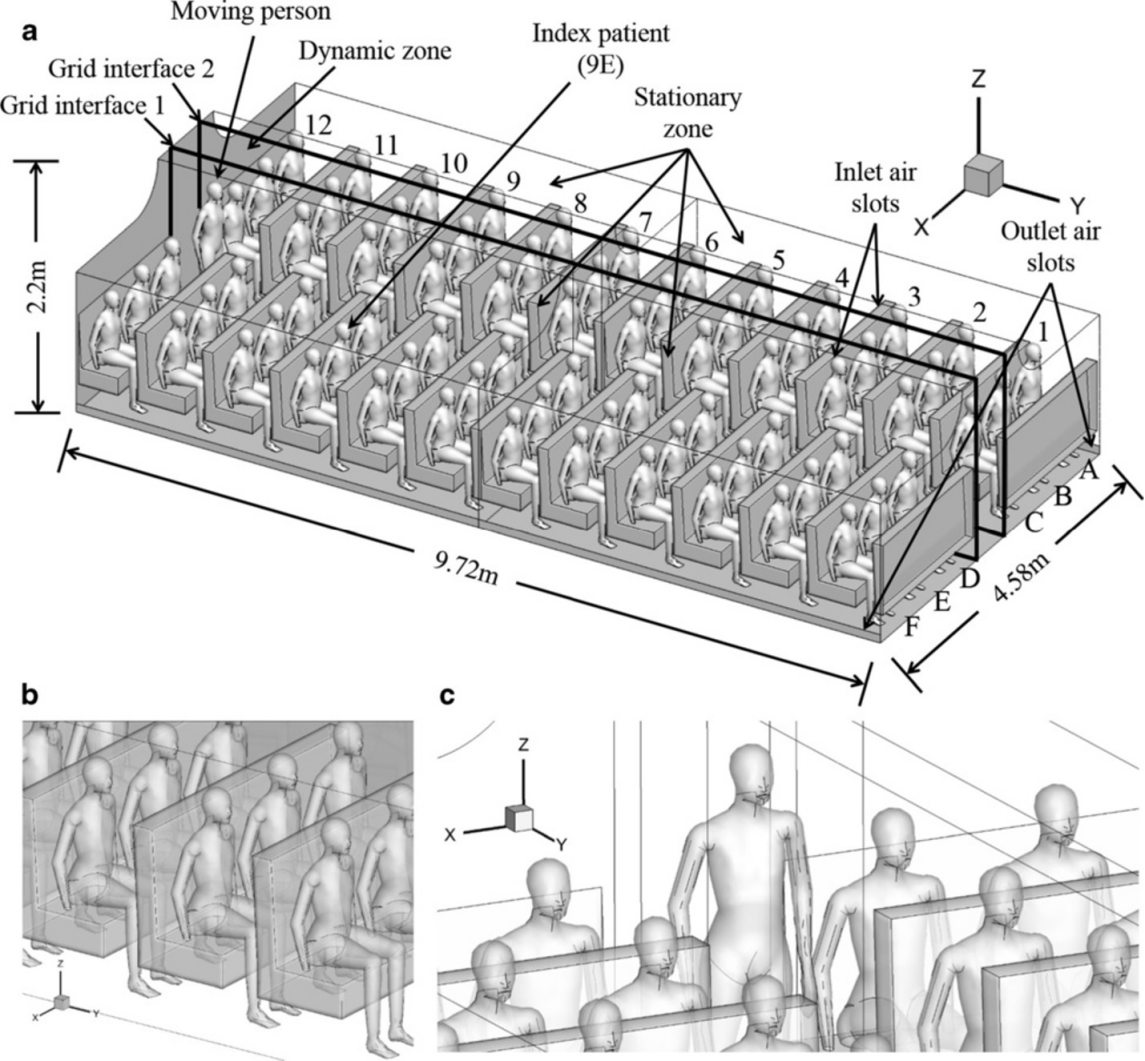
**Computational geometry of the airplane cabin with index patient seated at 9E. (a)** computational geometry of the airplane cabin, **(b)** detailed profiles of the seated passengers, **(c)** detailed profile of the moving person.

On March 15, 2003, 120 persons (112 passengers, 6 flight attendants and 2 pilots) flew from Hong Kong to Beijing by a Boeing 737–300, including an infector of SARS seated in the middle of the economy cabin of the airplane. After the three-hour flight, laboratory-confirmed SARS developed in 20 persons and 2 others gave diagnoses of probable SARS [6]. The computational geometry of the airplane cabin with occupants was numerically constructed and meshed based on the outbreak case. As shown in Figure 1(a), a twelve-row, single-aisle, fully occupied cabin section was constructed for simulation. Seventy-two passengers including the index patient were seated in the cabin. A movable person stood at one end of the aisle. The dimensions of the numerical airplane cabin section were 4.58 m × 9.72 m × 2.20 m (W × L × H) with a volume of 68.85 m^3^. The origin of the coordinate system was located on the ground at the left corner of the cabin behind the standing person, and the coordinate system met the right-hand rule. Ventilation air was supplied from two longitudinal overhead slots (0.012 m W × 9.72 m L each) located in the middle of the ceiling. The outlets were located along the lower longitudinal edge of the floor, one on each side. To represent the detailed profile of the occupants, a digital model was used, which was obtained by scanning a real thermal manikin using the 3D laser scanning system. Detailed facial features such as head, shoulders and limbs were contained in this model, as shown in Figure 1(b) and (c).

Gambit (version 2.4.6) was used to build the geometry domain and generate the cells for CFD simulation. The meshes were automatically generated by Gambit according to the mesh type and maximum mesh size. The whole region around the seated passengers and the standing person were meshed by unstructured grids of tetrahedron. The maximum mesh size was 0.03 m and the total number was 9,098,636. Other parts of the cabin (the aisle behind and in front of the standing person) were meshed by structured grids of hexahedron. The maximum mesh size was 0.025 m and the total number was 99,264. The grid system was chosen based on the grid convergence index (GCI) analysis [21]. By comparing the computed velocity magnitudes at 800 selected points in the GCI analysis, finer grid system did not have much improvement in GCI compared to the selected grid system (GCI_finer_ < 5%) [22]. So a grid system containing 9,197,900 cells was finally adopted in this computational geometry.

### Numerical approach

In this work, a multiphase numerical model based on the Eulerian–Lagrangian approach was adopted, which has been widely employed in aerosol dynamics simulation in enclosed environments [17, 22–24]. In this approach, the governing equations of the carrier phase were numerically solved in the computational geometry based on the Eulerian framework [23]. Transient species transport model for water vapor was added to the Eulerian carrier phase models to represent the humidity in the air [23]. For the discrete phase, the governing equations were described in the Lagrangian framework. Each aerosol released from the injections point was tracked individually in the Lagrangian frame for its instantaneous position and velocity.

The governing equations for the carrier phase and the discrete phase were solved by using a finite-volume based code, ANSYS (version 12.1.4). In the transient simulation, the interaction between the discrete phase and the continuous phase was also considered as the external body forces and computed during the continuous phase iterations [22]. The particles were tracked using the Lagrangian method along with the flow equations at the end of each time step. The Re-Normalization Group (RNG) k-ϵ model was used for modeling the turbulence in this computational geometry due to its good accuracy, computing efficiency, robustness and affordability [25–27]. The Differential Viscosity Model and the Swirl Dominated Flow in the RNG options were also used. An enhanced two-layer wall treatment was employed for the prediction of aerosol deposition [24]. Wall unit adaptation was applied in wall-adjacent cells when creating the meshes to ensure that the values of the wall unit y^+^ meet the requirements of the enhanced wall treatment [22]. The turbulent dispersion and the random walking of the aerosols are also considered by using the Thermophoretic Force and the Brownian Motion options in the DPM model. To simulate human movement, the layering meshing scheme of the dynamic mesh method was used [13, 17, 28]. The whole mesh domain was split into two mesh zones: stationary zone and dynamic zone [11], as shown in Figure 1. The surfaces between the static mesh zone and the dynamic mesh zone are set as grid interfaces. The grid sizes on the two sides of the interfaces are different. The maximum mesh size for the static mesh zones and the dynamic mesh zones is 0.03 m and 0.05 m, respectively. The data exchange in the interface between the static and dynamic mesh domains was realized by the grid interface principles for the sliding mesh theory [11]. The semi-implicit method for pressure-linked equations (SIMPLE) algorithm was employed to solve the pressure–velocity coupling equations in the steady-state. The Pressure-Implicit with Splitting of Operators (PISO) algorithm was employed for transient simulation. The second order upwind scheme was used for the treatment of the convection and diffusion-convection terms in the governing equation. The method used to simulate human movement has been verified by experimental and numerical investigations [29].

### Boundary conditions and case setup

The airflow pattern in this airplane cabin was first simulated in the steady-state. The inlet velocity was 2.994 m/s, corresponding to 9.7 L/s/person. The air exchange rate for the supply inlet conditions was 36.52ACH. The boundary conditions are shown in Table 2[8, 18, 19, 22]. The effects of the human thermal plume on the airflow motion was also considered and numerical simulated in the computational geometry. The surface temperature of the seated passengers was numerical set, following the method given by previous studies [18, 19, 22]. Since the inhalation and exhalation airflow velocity in the breathing zone of the person was small [30, 31], the influence of the respiration on aerosols transmission can be regarded as insignificant. So the personal respirations of the passengers were not simulated in this work [18]. For the respiratory activities of the index patient, breathing and cough is very common for SARS infector. According to previous studies, the droplets expelled by breathing are much smaller and fewer than that of coughing [32]. So in this work, coughing was considered as the main aerosol release process of the index patient, and the dissemination process of the coughing droplets were numerical simulated.

**Table 2:** **The boundary conditions in the numerical simulation**

Surface	Velocity	Temperature	Humidity ratio	Discrete phase
Ceiling	No slip	297 K	None	Trap
Side wall	No slip	293 K	None	Trap
Floor	No slip	296 K	None	Trap
Human body	No slip	305 K	None	Trap
Supply air	2.994 m/s	294 K	RH 20% (0.004895)	Reflect
	(9.7 L/s per person)			
Seat	No slip	Adiabatic	None	Trap
Outlet	Outflow			Escape
Nose and mouth of the index patient	10 m/s, t = 0-0.1 s	310.15 K	RH 50% (0.01224)	t = 0-0.4 s, Reflect
	6 m/s, t = 0.1-0.2 s			
	4 m/s, t = 0.2-0.3 s			
	2 m/s, t = 0.3-0.4 s			t > 0.4 s, Trap
	0 m/s, t > 0.4 s			
	Open area: 0.000968 m^2^			
Back and front surface	Periodic			Escape

To simulate a cough, aerosol injections were employed right at the mouth of the index patient. According to experimental measurements of cough droplets, the quantity of the droplets smaller than 3 μm was quite small, and the droplets larger than 100 μm might fall down to the ground immediately after they were expelled [33, 34]. So in this work, the original size range of the aerosols was 3 μm ~ 112 μm and ten size classes were used to simulate the size distribution. In enclosed environment, the evaporation effects will strongly influence the size and mass of the droplets [32]. Considering that the equilibrium diameter after evaporation was 50% of the original size [35], the size range of the aerosol injections was 1.5 ~ 56 μm and the size distribution followed the experimental results given by Duguid [34]. The particles number of each size class was set as 50,000 and the total number of the particles of the ten size classes was 500, 000, follows the aerosol setup used by previous studies [18, 22]. The total volume of this computational geometry is larger than that of Reference [22] (30 m^3^) and smaller than that of Reference [18] (97 m^3^), thus the total number of the particles used in this numerical simulation can be considered as enough for this study. This number would be converted to the original number according to the size distribution of cough for infection risk assessment. The size distribution of the droplets used in the numerical simulation is shown in Table 3. The coughing direction was 45 degree downwards (0, 1, −1). When the index patient coughs, the open area of his mouth was 9.68 cm^2^. The velocity of the exhaled airflow was set according to the experimental results to approximately simulate a cough [19].

**Table 3:** **Size distribution of the aerosol injections**

Diameter (μm)	Original size (μm)	Total number	Size distribution [34]
1.5	3	50000	0.0105
3	6	50000	0.0610
6	12	50000	0.2040
10	20	50000	0.3365
14	28	50000	0.1830
18	36	50000	0.0883
22.5	45	50000	0.0463
31.25	62.5	50000	0.0231
43.75	87.5	50000	0.0294
56	112	50000	0.0179

In this airplane section, the airflow pattern in the steady-state was simulated and used as the initial condition. Then, five cases under different human movement pattern assumptions were investigated. Case 1 ~ 3 were used to simulate the cough when the index patient was seated at 9E, which is also the injection location. A no-movement case was used to simulate the aerosol dispersion in the airplane cabin without human movement. Two more cases were simulated to study the aerodynamic effects of human movement, in which the standing person started to move along the aisle 1 s after the aerosol injections at moving speeds of 0.5 m/s and 1.0 m/s, corresponding to stroll and normal walking, respectively. Case 4 and 5 simulated and compared the aerosol dispersion when the index patient walked in the airplane at 0.5 m/s and coughed at 3 s and 6 s after the movement began, respectively. Since the aisle in the Boeing airplane is much longer than this computational geometry and the moving person may moves from a further area outside this computational geometry, the walking speed of the moving person can be assumed remain the same during the movement in this computational geometry [9]. The walking speed profile of the moving person is an even-speed profile [8]. The investigated cases are given in Table 4.

**Table 4:** **The cases investigated in the numerical simulation**

Case no.	Injection person	Injection time	Movement start time (s)	Moving speed (m/s)
1	Index person at 9E	t = 0 s	\	0
2	Index person at 9E	t = 0 s	t = 1 s	0.5
3	Index person at 9E	t = 0 s	t = 1 s	1.0
4	Moving person	t = 3 s	t = 0 s	0.5
5	Moving person	t = 6 s	t = 0 s	0.5

In the numerical simulation, the time step was 0.1 s for t ≤ 10s and 0.2 s for t > 10s when the standing person was not moving. During human movement, the time step was 0.1 s for moving speed of 0.5 m/s and 0.05 s for 1.0 m/s. Each case was computed in a 4-node Linux cluster. Each node of the cluster had eight processors (2.4 GHz Intel 64) and 16 GB of memory. The calculation time of each case was 180–220 hours, depending on the total number of time steps and iterations.

### Risk assessment and likelihood analysis

Based on the results of CFD simulation, the infection risks of the occupants were investigated by using the dose–response model in the risk assessment [36]. The exposure levels of the passengers were assessed according to the concept of intake fraction [18, 37], which demonstrated the fraction of the quantity of pathogens deposited on the target infection site in the respiratory tract to the total quantity of pathogens produced by the index patient [38]:

1 $$D\left(x,t\right)=\frac{\sum_{l=1}^{m}\beta_{l}\mathrm{cp}\int v{\left(x,t\right)}_{l}h_{l}f\left(t\right)\mathrm{dt}}{N_{c}}$$

where *D*(*x*,*t*) is the intake fraction of the susceptible passengers for one cough. *x* is the spatial location *c* is the pathogen concentration in the expiratory fluid, 10^6^*pfu*/*ml* for the SARS-CoV [39]. *p* is the pulmonary ventilation rate, 7.5 *l*/min [18]. *f*(*t*) is the viability function of pathogens in the aerosols. Since the SARS-CoV may retain its infectivity for minutes and gradual loss for as long as several days [40, 41], the viability function of the SARS-CoV is taken as 75% after aerosolization and remains the same during the first a few minutes [22, 40, 41]. *m* is the total number of size bins. *N*_*c*_ is the total quantity of pathogens produced in a cough, *N*_*c*_ = *V*_*c*_*c*, where *V*_*c*_ is the total volume of the droplets produced in a cough, 6.7 × 10^-3^*ml*[18, 42]*h*_*l*_ is the ratio of the number of droplets of the *l*th size bin in a cough to the number of injected particles in the numerical model, in which the number of droplets of the *l*th size bin can be calculated according to the original size distribution of the cough and *V*_*c*_. *v*(*x,t*) is the volume density of expiratory droplets in the breathing zone of the subject induced by one cough, *ml*/*l* of air. The breathing zone of each passenger can be defined as a hemisphere with 0.3 m radius at the nose [18]. For the human model used in this work, the volume of the breathing zone is 0.005721 *m*^3^ for each passenger. Then *v*(*x,t*) can be calculated according to the CFD simulation results as the total volume of all the expiratory droplets in the breathing zone divided by the volume of the breathing zone [43]. *β*_*l*_ is the respiratory deposition fraction of the aerosols of the *l*th size bin, %, which can be calculated according to the size and deposition location of the aerosols [44]. According to the infection mechanism of SARS, the human angiotensin 1-converting enzyme 2 (hACE2) has been confirmed to be the receptor of the SARS-CoV. The hACE2 can be detected in ciliated airway epithelial cells of human airway tissues derived from nasal and tracheobronchial regions. And the infectivity of the SARS-CoV on the ciliated airway epithelial cells derived from nasal and tracheobronchial regions shows no difference [45]. So the aerosols that deposit in the head airway and tracheobronchial regions of the respiratory tract can be accounted in the intake dose [44].

It was tedious and time-consuming to model every cough during the exposure time interval to obtain *v*(*x,t*) at different locations. Considering other aerodynamic size-dependent factors, a stochastic non-threshold dose–response model for airborne pathogens can be formed [38]:

2 $$\begin{array}{l} P_{I}\left(x,t_{0}\right)=1-\exp\left(-\sum_{l=1}^{m}r_{l}\beta_{l}f_{s}t_{0}\mathrm{cp}\int_{0}^{t_{0}}v{\left(x,t\right)}_{l}h_{l}f\left(t\right)\mathrm{dt}\right)\\ \;=1-\exp\left(-rN_{c}f_{s}t_{0}D\left(x,t_{0}\right)\right) \end{array}$$

where *P*_*I*_ is the infection risk of the susceptible passenger after the flight, which demonstrates the infection possibility of the susceptible passenger; *t*_*0*_ is the exposure time interval of the flight, *hr*; *f*_*s*_ is the cough frequency, 18/hr [46]; *r*_*l*_ is the infectivity of pathogens in the droplets of the *l*th size bin; and *r* is the integrated infectivity factor for all pathogens. For the SARS-CoV, the infectivity of pathogens contained in the droplets of different size classes show no differences, hence the infectivity *r*_*l*_ can be expressed as *r*. According to the experiments on mice for the development of vaccine, efficient replication of the virus is found in the respiratory tract of the mice after they are administered at a very low dose of the SARS-CoV [47, 48], indicating high infectivity of the SARS-CoV. Since no other infectivity data is found, the infectivity factor *r* is assumed as 1 per pfu of virus in this work because of the high infectivity of the SARS-CoV. In Eq. (2), *N*_*c*_*f*_*s*_*t*_0_*D*(*x*, *t*_0_) can also be regarded as the intake dose of the passenger.

To evaluate the effects of different human movement behaviors, the intake fractions of the seated passengers can be expressed as followed:

3 $$D\left(x,t\right)=\sum_{q}a_{q}D_{q}\left(x,t\right)$$

where *D*(*x*,*t*) is the intake fraction of the susceptible passenger, which is defined in Eq. (1) and used in Eq. (2); *D*_*q*_(*x*,*t*) is the intake fraction of the susceptible passenger for the *q*th movement behavior; and *a*_*q*_ is the possibility of the *q*th movement behavior; $\sum_{q}a_{q}=1$.

In the risk assessment approach, average or assumed values were used for the input parameters of Eq. (1) and (2), e.g. the pathogen generation rate of the index patient. In the actual outbreak case, the values of these parameters may be different from the average values because of the individual differences of the index patients. These differences may significantly increase the uncertainty of the risk assessment results. So the likelihood analysis was performed as a hypothesis test on the input parameters and assess which human movement pattern assumption was most likely to be the actual case [49]. During the estimation of likelihood, the uncertainties of the unknown parameters were all considered in the quanta generation rate. The quanta generation rate was the generation rate of the infective pathogens, which can be regarded as the multiple of the infectivity factor to the pathogen generation rate (*rN*_*c*_*f*_*s*_, *hr*^− 1^), as described in Sze To & Chao [36]. While the risk assessment provided quantitative information on how did human movement affect the infection risks of the passengers, the likelihood analysis served as a better tool to estimate the unknown information in the actual outbreak case.

In the likelihood analysis, all the susceptible passengers were divided into several groups, and passengers with similar intake fractions were grouped in the same group. Maximum likelihood estimation (MLE) was then determined to identify the value of quanta generation rate that was most likely to be the true value [49]. The case with the MLE can be regarded as the case that best fits the outcome of the outbreak. The likelihood can be estimated as followed:

4 $$\begin{array}{l} \bar{L_{r}\left(p_{I}\right)}={\left(\prod_{s=1}^{S}N_{s}L_{r}\left(\bar{p_{I,s}}\right)\right)}^{1/S}\\ \mathrm{where}\;L_{r}\left(\bar{p_{I,s}}\right)\left\{\begin{array}{c} \;{\left(1-\bar{p_{I,s}}\right)}^{{}^{N_{s}}}\;\mathrm{for}\;n_{s}=0\\ \;{\bar{p_{{}_{I,s}}}}^{N_{s}}\;\mathrm{for}\;n_{s}=N_{s}\\ {N_{s}}^{N_{s}}{\left(\frac{\bar{p_{I,s}}}{n_{s}}\right)}^{n_{s}}{\left[\frac{\left(1-\bar{p_{I,s}}\right)}{N_{s}-n_{s}}\right]}^{N_{s}-n_{s}}\;\mathrm{for}\;\mathrm{other}\;n_{s} \end{array}\right. \end{array}$$

where $\bar{L_{r}\left(p_{I}\right)}$ is the average relative likelihood; *S* is the total number of the divided groups; *N*_*s*_ is the total number of susceptible people in the sth group; $L_{r}\left(\bar{p_{I,s}}\right)$ is the relative likelihood of the sth group; $\bar{p_{I,s}}$ is the average infection risk of the sth group; and *n*_s_ is number of infected people in the sth group.

## Results

### Airflow pattern

In this work, the steady-state airflow pattern in the cabin was used as the initial condition for the transient simulation. In steady-state, the cold air comes into the airplane cabin from the supply inlets, flows downward and sideward through the cabin and exits from the outlets located at the bottom of the sidewalls. No symmetrical recirculation zone exists, unlike the airflow pattern in the twin-aisle airplane [18, 19, 22]. Downward airflow can be found on both sides of the aisle, which is induced by the ventilation system. The human thermal plume is not apparent because of the significant downward airflow induced by the ventilation system. The heat effects of the human body can influence the temperature distribution in the local area around the human body. The influence area of these downward airflow is also larger than that of the respiratory exhalation flows of the seated passengers, which indicates that the influence of the personal respiratory exhalation flows of the seated passengers on the airflow field is insignificant comparing with that induced by the ventilation system.

When the standing person moves along the aisle, the airflow pattern in the airplane is affected by human movement, similar to the results given by previous studies [8, 11]. Significant downward airflow exists in the wake behind the torso, which enhances the downward movement of the room air induced by the ventilation system. Hence human movement can disturb the air distribution in a local region and influence the airflow motion in the airplane cabin. More details about the airflow pattern in the airplane cabin, and discussions on the human thermal plume and the effects of human movement on the flow field can be found in the Additional file 1.

### Aerosol dispersion and deposition

**Figure 2:**
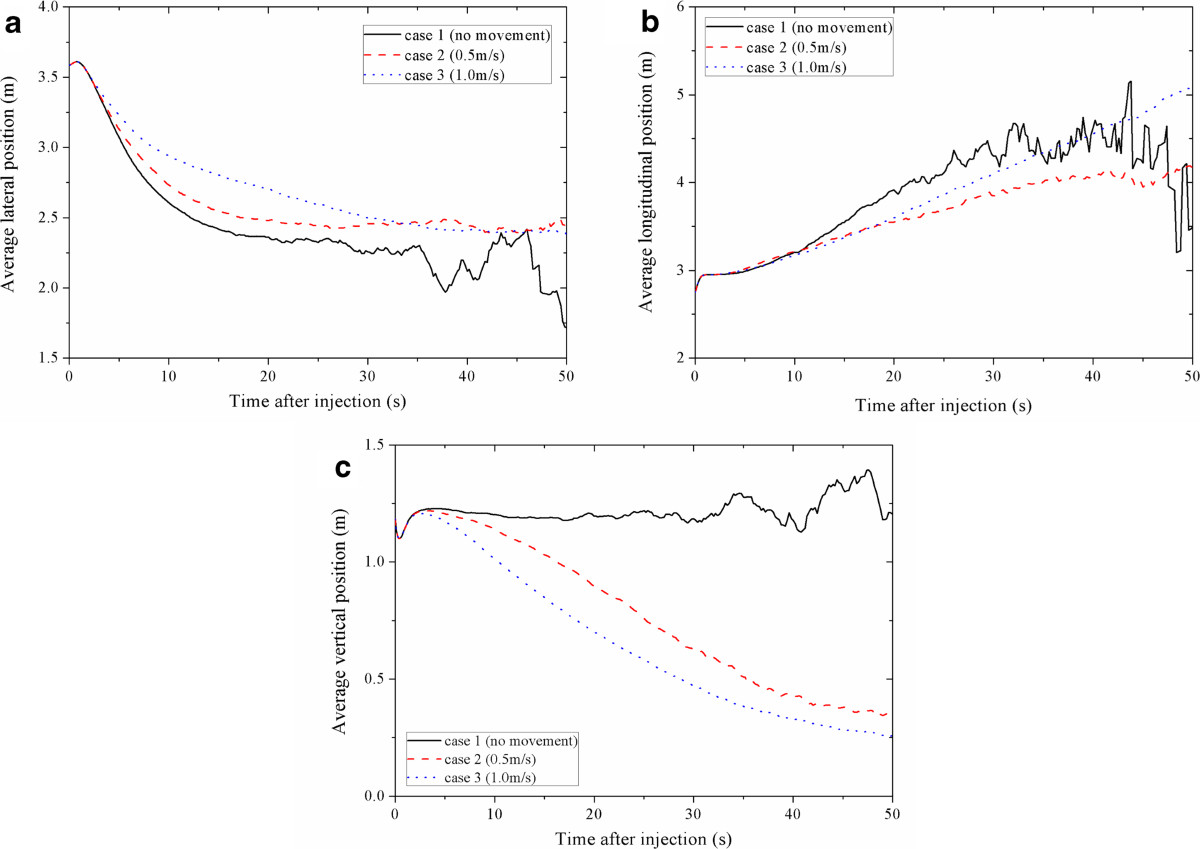
**Average position of all the aerosols for case 1 ~ 3. (a)** X direction, **(b)** Y direction, **(c)** Z direction.

To demonstrate the effects of human movement on aerosol dispersion in the airplane cabin, Figure 2 gives the timing diagrams of the average position of all the aerosols for case 1 ~ 3 in X direction (lateral position), Y direction (longitudinal position) and Z direction (vertical position), respectively. The average position is the average value of the absolute positions of all the airborne aerosols at time t.

From Figure 2(a), similar trends can be found for all cases. The average lateral positions first increase to 3.61 m in 0.8 s after the injections and then keep decreasing. Higher moving speeds lead to a slower decreasing rate. In the computational geometry, the index patient was seated at X = 3.585 m and the position of the centerline was X = 2.29 m. So Figure 2(a) suggest that the airflow induced by human movement will slightly prevent the aerosols from moving across the aisle of the airplane cabin. Figure 2(b) shows that the average longitudinal position keeps increasing after the injections for case 1 ~ 3. Since the airflow from front to back of the cabin (longitudinal direction) is minimal [6, 7], the longitudinal movement of the aerosols of case 1 is mainly due to the initial momentum given by the cough. Figure 2(b) also indicates that human movement may enhance the mixing of room air in the airplane cabin, which may result in the transport of the aerosols along the moving path in both the positive and negative longitudinal direction, not only following the coughing direction or the moving direction. This is also proven by the results of Exposure assessment shown in Section 3.3, in which the aerosol concentrations in the breathing zones of the passengers who are seated ahead and behind the index patient will increase after the movement of the standing person. As shown in Figure 2(c), the average vertical position of case 1 remains at about 1.20 m and starts to fluctuate after 25 s. But for case 2 and 3, it keeps decreasing and is lower than 0.5 m after 40 s. So human movement may cause the suspended aerosols to fall down to the ground and result in a downward transport effect. During human walking, apparent downwash flow and downward contaminant transport can be found in the wake behind the torso of the human body [9]. Thus the downward transport of the aerosols may be mainly due to flow characteristics of the wake behind the human body. In an airplane cabin, not only the ventilation system but also the wake of a moving human can strengthen the downward transport of the aerosols. Higher moving speed may have a stronger downward transport effect.

**Figure 3:**
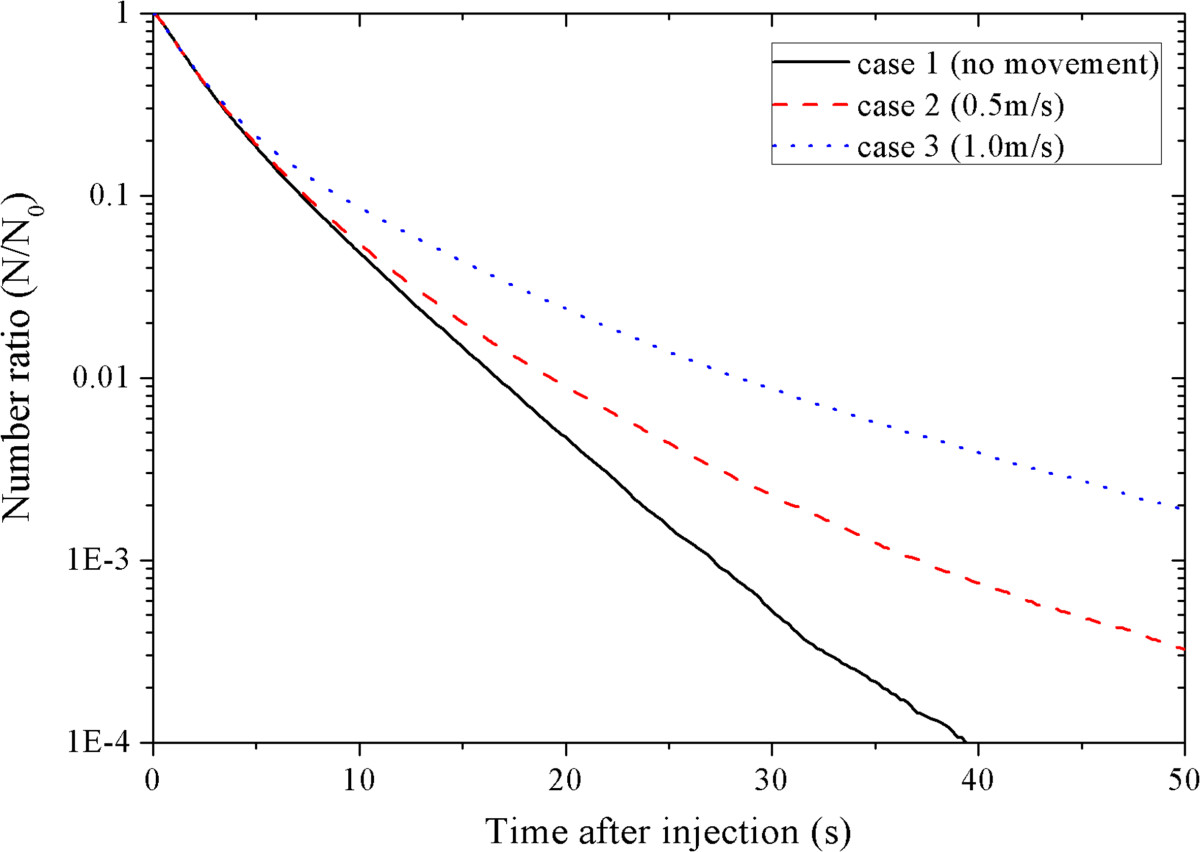
**Comparison of the suspended fraction of all the aerosols for different cases.**

From Figure 2(a) ~ (c), apparent and fierce fluctuation trend of the average position can be found for case 1 in all of the three directions when t > 30 s. Fluctuation can also be found for case 2 after t > 40 s, which is not as fierce as that of case 1. This fluctuation is because the number of the suspended aerosols is too small [22]. Hence Figure 2 also suggests that human movement may influence the number of the suspended aerosols as well as the deposition and removal of the aerosols. To demonstrate the effects of human movement on deposition and removal of the aerosols, Figure 3 shows the suspended fraction of all the aerosols for case 1 ~ 3. This suspended fraction is the ratio of the number of the aerosols remained airborne to the total number of injected aerosols. As shown in Figure 3, the suspended fractions of case 1 ~ 3 keep decreasing. For higher moving speed, the suspended fraction decreases more slowly. For case 1, when t ≥ 27.2 s, the number of the aerosols remained airborne is less than 500 and the suspended fraction is less than 10^−3^. When t ≥ 39.6 s, the suspended fraction is less than 10^−4^. For case 2, the number of the aerosols remained airborne is larger than 500 until t = 37.2 s and the suspended fraction remains larger than 10^−4^ even after 50 s after the injections. For case 3, there are still more than 900 aerosols remained airborne after 50 s after the injections, and the suspended fraction remains larger than 10^−3^. This result indicates that the turbulence effects, which are induced by the movement of the standing person, can significantly lower the deposition and removal rate of the aerosols and influence the fluctuation of the average positions of the suspended aerosols. In case 1 ~ 3, more than 99% of the aerosols are deposited in the cabin. So the movement of the standing person does not significantly change the total deposition fraction of the aerosols.

**Figure 4:**
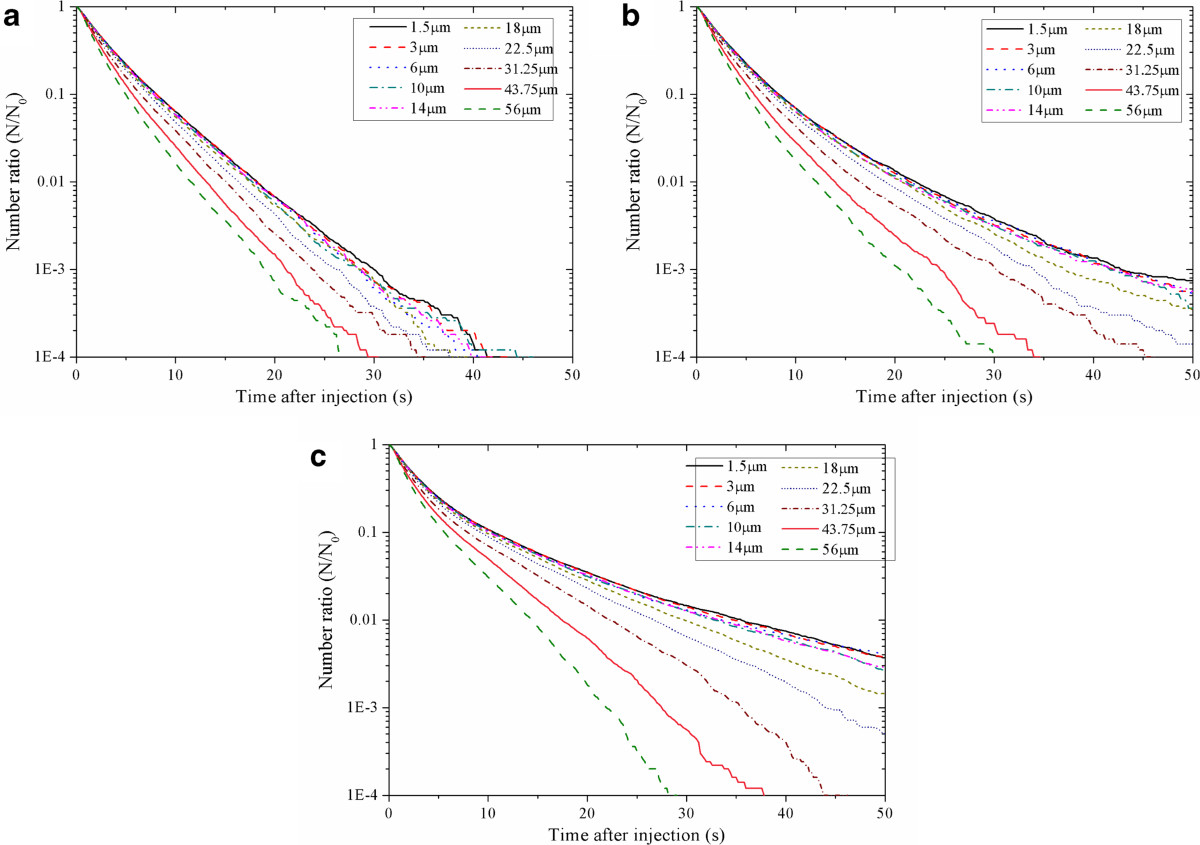
**Relationship between suspended fraction and aerosol size for case 1 ~ 3. (a)** case 1 (no human movement), **(b)** case 2 (moving speed: 0.5 m/s), **(c)** case 3 (moving speed: 1.0 m/s).

To demonstrate the effects of human movement on the deposition and removal of the aerosols of different size, Figure 4 gives the relationship between suspended fraction and aerosol size for case 1 ~ 3, respectively. Similar to Figure 3, Figure 4 shows that the suspended fractions of all aerosol sizes decrease quickly. For case 1, at 30 s after the injections, the suspended fractions of all aerosol sizes are smaller than 10^−3^. For case 2, the suspended fractions of the aerosols smaller than 18 μm remain larger than 10^−3^ until 43 s after the injections. For case 3, the suspended fractions of the aerosols smaller than 22 μm are still larger than 10^−3^ even after 50 s after the injections. So the movement of the standing person can significantly lower the deposition and removal rates of all aerosol sizes. From Figure 4(a) ~ (c), similarity can also be found between the characteristics of the deposition and removal rates of different aerosol sizes. The suspended fractions of larger aerosols decrease more quickly than that of smaller aerosols. These characteristics are not affected by human movement.

### Exposure assessment

**Figure 5:**
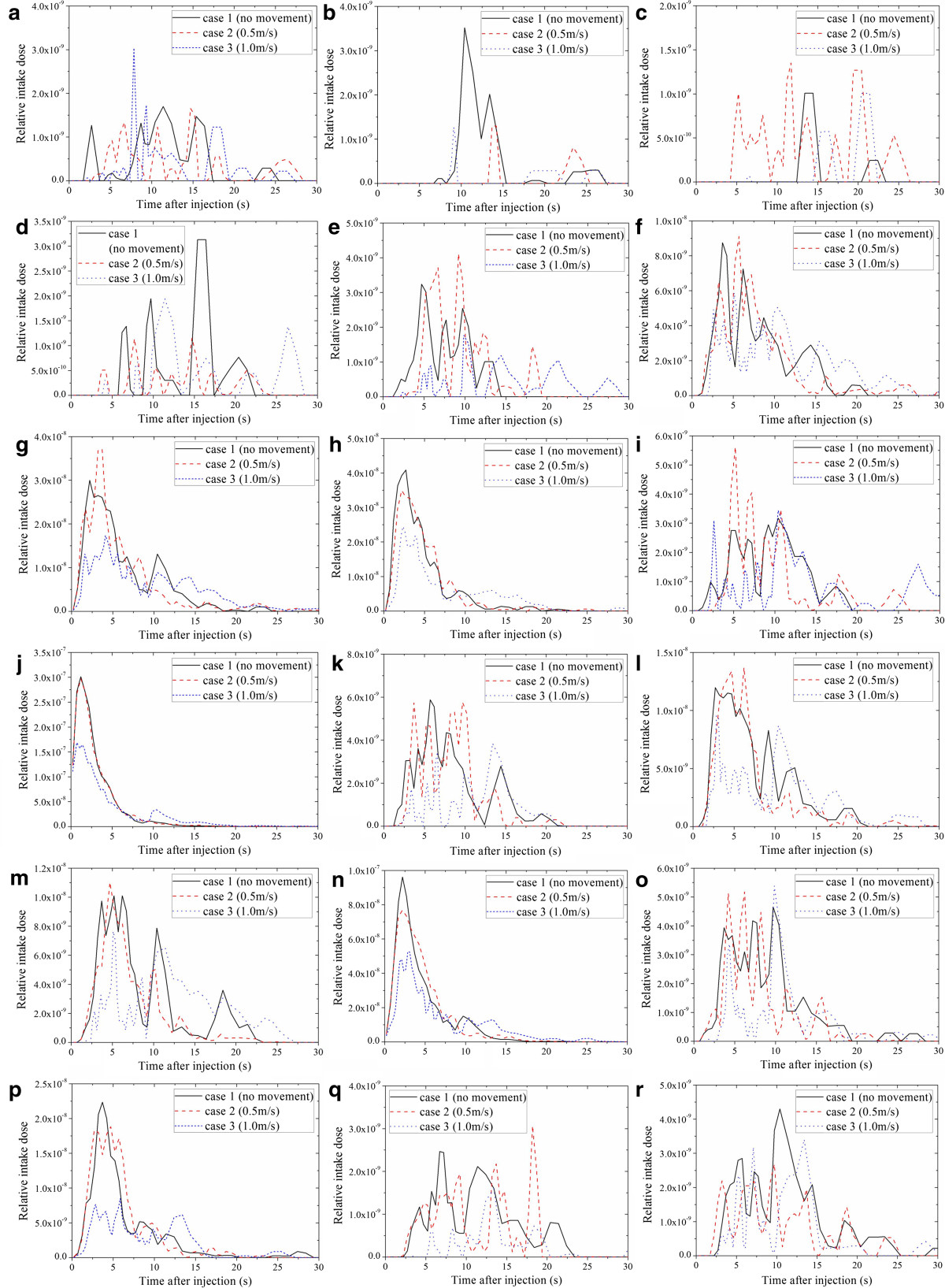
**Time profiles of the relative intake dose of the passengers for case 1 ~ 3. (a)** passenger 1D, **(b)** passenger 2C, **(c)** passenger 3E, **(d)** passenger 4C, **(e)** passenger 5E, **(f)** passenger 6D, **(g)** passenger 7D, **(h)** passenger 7 F, **(i)** passenger 8A, **(j)** passenger 8E, **(k)** passenger 9A, **(l)** passenger 9B, **(m)** passenger 10B, **(n)** passenger 10D, **(o)** passenger 11B, **(p)** passenger 11 F, **(q)** passenger 12B, **(r)** passenger 12C.

Based on the simulation results, the number of expiratory droplets in the breathing zone of each passenger can be obtained according to the locations of the suspended aerosols and the breathing zone. Then the volume density of expiratory droplets *v*(*x*,*t*) can be calculated and the intake fraction can also be obtained by using Eq. (1). Figure 5 shows the exposure levels of eighteen selected passengers from line 1 ~ 12 and seat A ~ F for case 1 ~ 3, including seven passengers infected with SARS after the flight (passenger 2C, 5E, 7D, 8E, 9B, 11B and 12B) and eleven healthy passengers (passenger 1D, 3E, 4C, 6D, 7 F, 8A, 9A, 10B, 10D, 11 F and 12C). The exposure level is demonstrated by the relative intake dose, which is the intake fraction of the passenger in each time step. The seating plan of the passengers can be found in Figure 1. Figure 5 shows that the relative intake dose of each passenger is significantly different for different moving speeds. Even the passengers seated 8 rows ahead or 3 rows behind the index patient are affected by the movement. Hence the volume density distribution of expiratory droplets in the airplane cabin and the relative intake dose of each passenger can be significantly influenced by human movement.

**Figure 6:**
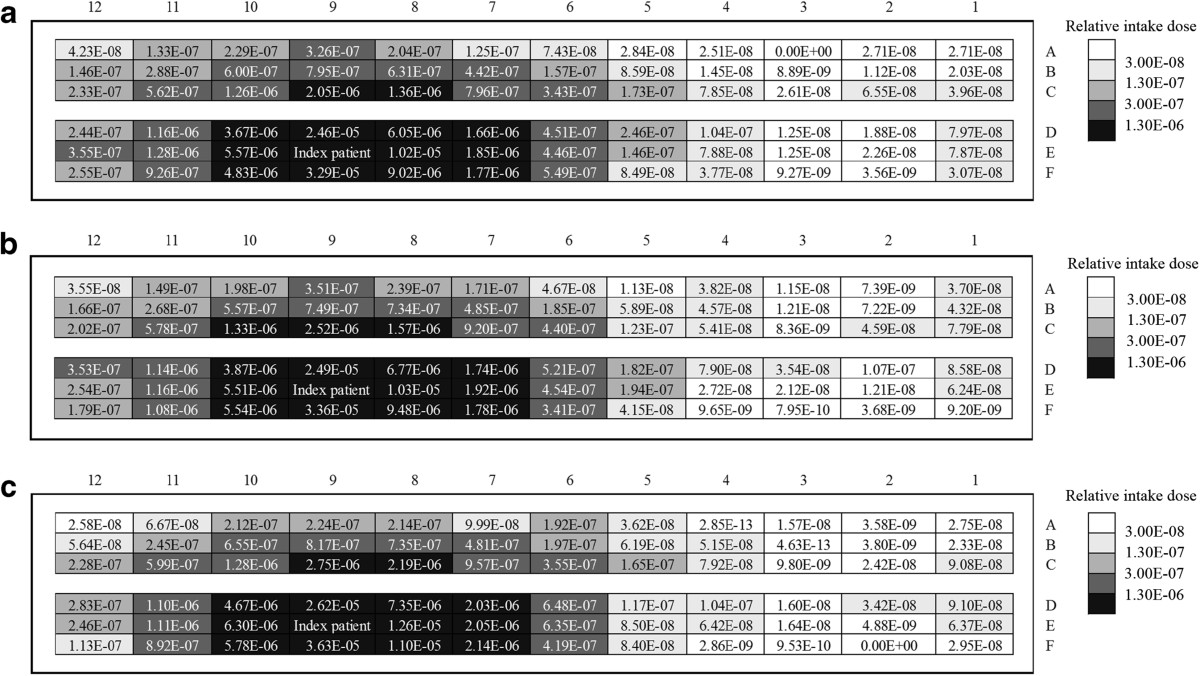
**Intake fraction of each passenger for case 1 ~ 3. (a)** case 1 (no human movement), **(b)** case 2 (0.5 m/s), **(c)** case 3 (1.0 m/s).

**Figure 7:**
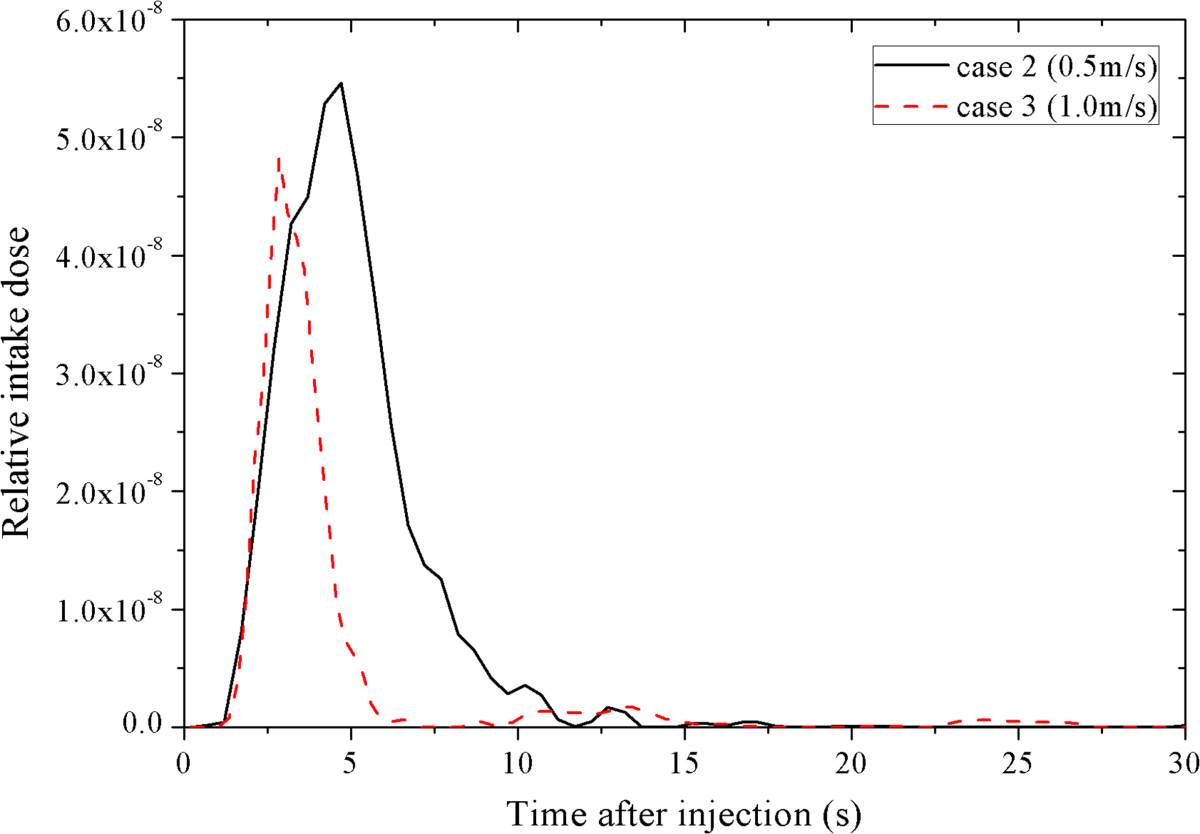
**Time profiles of the relative intake dose of the moving person at moving speeds of 0.5 m/s and 1.0 m/s.**

Figure 6 demonstrates the intake fraction distribution induced by one cough in the airplane cabin for case 1 ~ 3, respectively. Passengers who are seated close to the index patient (especially for row 7–10, seat D-F) have much higher intake fractions than other passengers. The intake fractions of the passengers seated on the right side of the moving person (seat D-F) are slightly higher than that on the left side (seat A-C). Lower intake fractions can be found from the passengers seated far away from the index patient (row 1–4). Comparing Figure 6(a) ~ (c), differences can be found in the intake fraction of every passenger. After human movement, more than half of the passengers will have higher intake fractions (41 passengers for case 2 and 38 for case 3). Compared with case 1, the variation range of the intake fractions is −91% ~ +472% for case 2 and −99% ~ +255% for case 3. The average intake fraction of all the 71 seated passenger (with the index patient and the moving person excluded) is 1.69 × 10^−6^, 1.75 × 10^−6^ and 1.91 × 10^−6^ for case 1 ~ 3. That means the movement of the standing person may lead to a 3.3% ~ 9.3% increase in the average intake fraction. Besides, some of the passengers seated 2–3 rows behind and 4–7 rows in front of the index patient may also have lower intake fractions after the movement. Thus the variation of the intake fractions also depends on the location of the passengers and the index patient. Besides, although the intake fractions of the passengers are changed by the movement of the standing person, no significant differences can be found between the distribution characteristics of the intake fractions of different cases. Passengers seated farther away from the index patient have much lower intake fractions.The walking person also has a non-negligible exposure level. Figure 7 gives a thirty-second time profile of the relative intake dose of the moving person for moving speeds of 0.5 m/s and 1.0 m/s (case 2 and 3). As shown in Figure 7, the relative intake dose of the moving person increases quickly after the person starts to move (t ≥ 1 s). The peak value appears at t = 5 s and t = 3 s for case 2 and 3, corresponding to a moving distance of 2 m, where the moving person is quite close to the index patient. Figure 7 also indicates a high relative intake dose during the movement (t = 2.5 ~ 7 s for 0.5 m/s and t = 2 ~ 5 s for 1.0 m/s), when the moving person walks through the region with high aerosol concentration (row 6–11). So a passenger or a crew member will receive a high intake dose if he/she walks through the aisle when the index patient is coughing.

### Risk assessment and likelihood analysis

**Figure 8:**
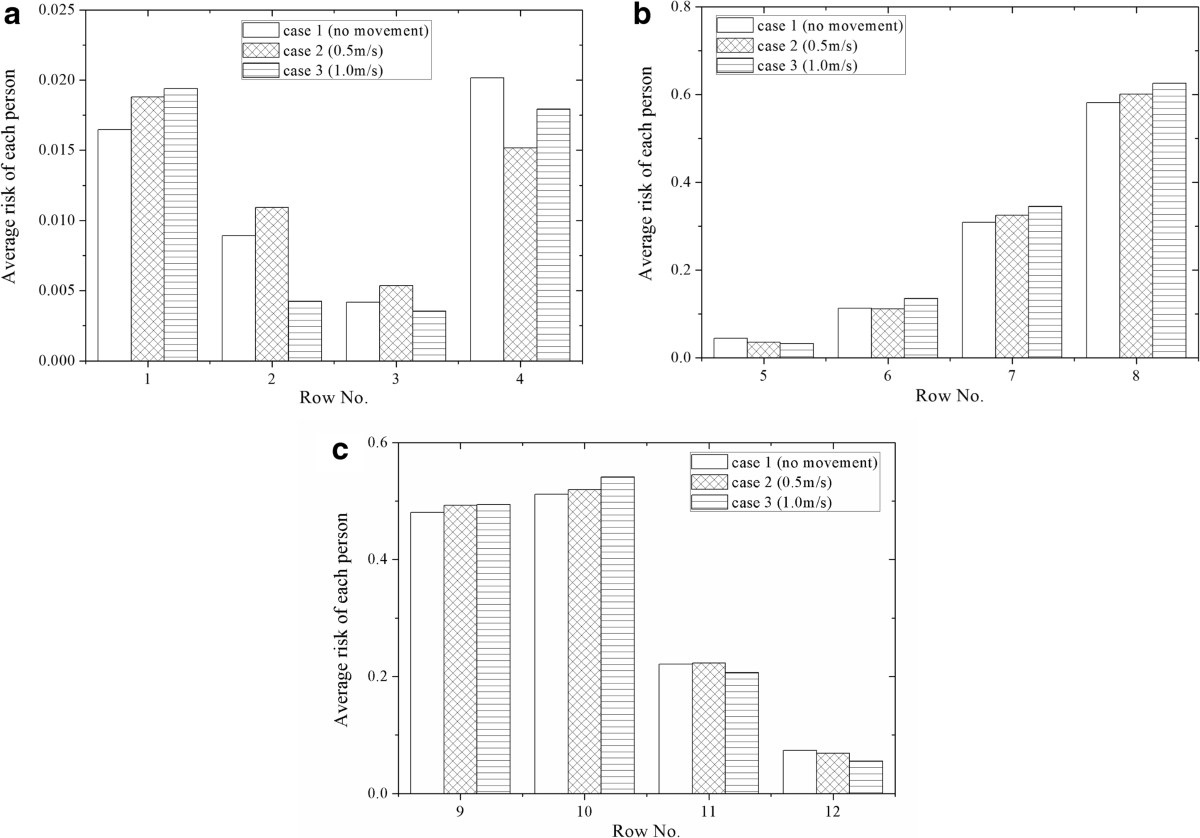
**Average infection risk per passenger of each row for case 1 ~ 3 (with 9E excluded). (a)** row 1~4, **(b)** row 5~8, **(c)** row 9~12.

**Figure 9:**
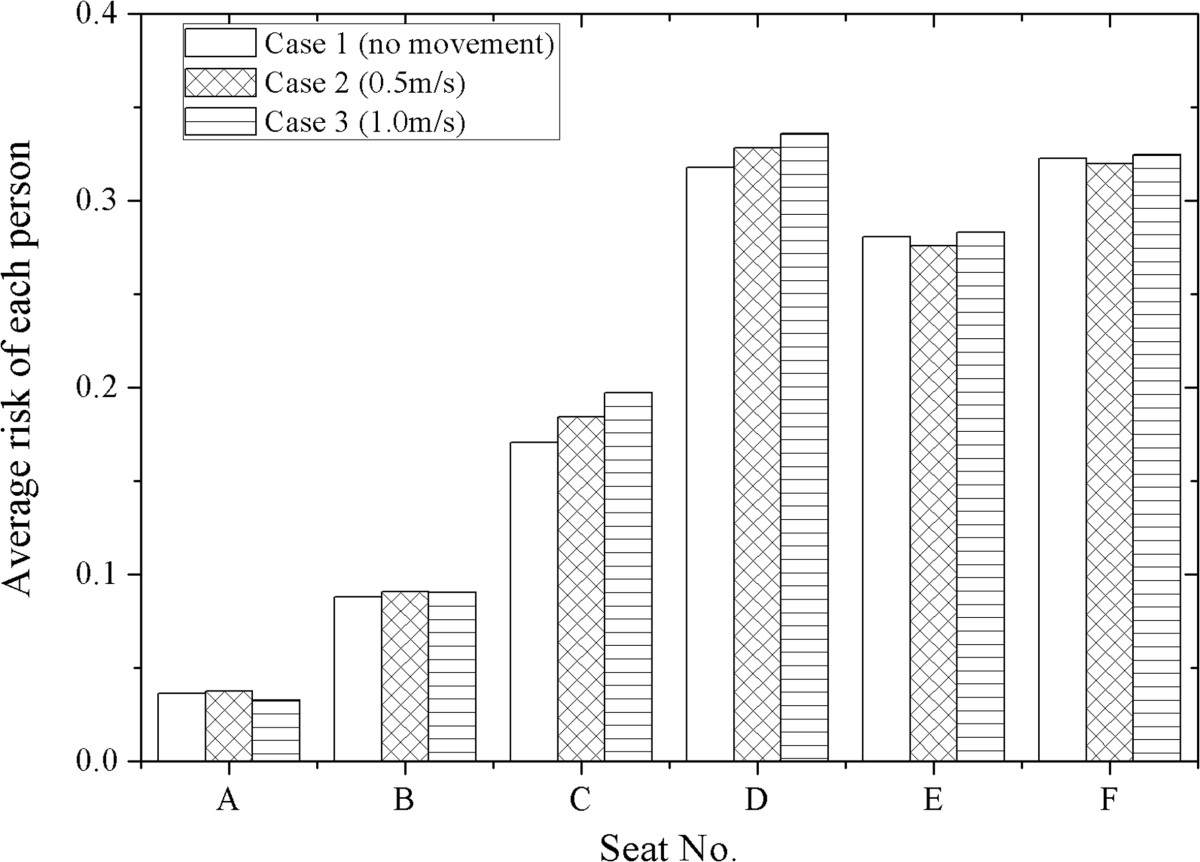
**Average infection risk per passenger of each line of seats for case 1 ~ 3 (with 9E excluded).**

By using the risk assessment, the influence of human movement on infectious disease transmission can be analyzed and compared. According to Eq. (2), the infection risk of each passenger after the 3-hour flight can be calculated for case 1 ~ 3. For case 2 and 3, the infection risk is calculated under the assumption that the moving person walks in the airplane frequently during the flight and he/she will walk through the aisle during each cough of the index patient.Figures 8 and 9 demonstrate the average infection risk per passenger in the airplane cabin for case 1 ~ 3. Figure 8 is the average of the infection risk of the six passengers seated in the same row (row 1 ~ 12), and Figure 9 is the average of the twelve passengers seated in the same line of seats (seat A ~ F). In Figures 8 and 9, the calculated infection risk of the index patient is excluded since he is already infected. As shown in Figure 8, the infection risks of the passengers close to the index patient (row 8–10) are much higher than others. Further area has a lower infection risk, except for row 1 and 2 where the moving person stops. From Figure 8, it also can be seen that the average infection risk of each row is affected by human movement. The movement of the standing person increases the average infection risk in row 6–8 (three rows in front of the index patient), row 9 (the same row of the index patient), row 10 (one row behind the index patient) and row 1 (where the moving person stops). For other areas, whether the movement can increase the infection risk depends on the moving speed. As shown in Figure 9, the average infection risk of the passengers in seat B ~ D will increase after human movement. The average infection risk of the passengers in seat A will also increase when the moving speed is 0.5 m/s. This result indicates that human movement may influence the infection risk distribution in the airplane cabin and lead to increases in the infection risks of more than half of the passengers.

With the moving person and the index patient excluded, the average infection risk of all the seated passengers is 0.2015, 0.2051 and 0.2096 for case 1 ~ 3, respectively. This result also proves that human movement may increase the infection risk in the airplane cabin. Especially, in case 2 and 3, the infection risk of the moving person is 0.55 for moving speed of 0.5 m/s and 0.51 for 1.0 m/s, significantly higher than most of the seated passengers (62 passengers for case 2 and 58 for case 3). This result indicates that the infection possibilities of the crew members (who need to walk in the airplane cabin frequently during the flight) may be even higher than 50%. For the seated passengers, walking along the aisle (e.g. go to the washroom) and passing through the high-dose region (around the index patient) may also significantly increase their intake doses and result in much higher infection risks.

**Figure 10:**
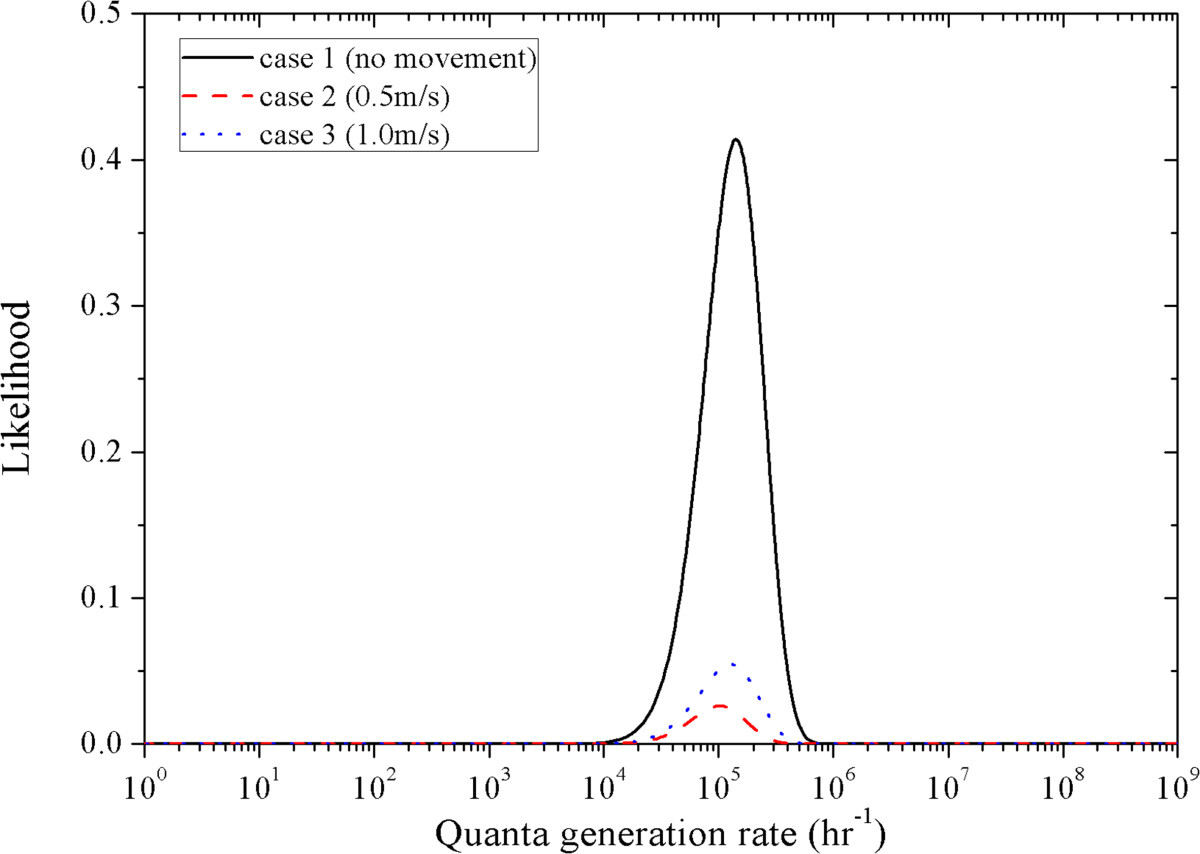
**Likelihood values under a range of quanta generation rates for case 1 ~ 3.**

In the risk assessment, an assumed value of 1 was used for the infectivity of SARS-CoV, and average values were used for the total quantity of pathogens produced in a cough. These average or assumed values might lead to uncertainties in the risk assessment results. In the likelihood analysis, all these uncertain variables were included in the quanta generation rate term, while the case that best fits the outcome of the real outbreak case was identified by the MLE [49]. As shown in Table 5, the passengers are arranged into four groups (with the index patient excluded) according to their intake fractions obtained in the exposure assessment of case 1 ~ 3 (Figure 6), respectively. In the outbreak case, 17 of the 71 passengers involved in this investigation region are infected with SARS. They are also given in Table 5 and used in the likelihood analysis. By using Eq. (4), the likelihood values under a range of quanta generation rates (1 ~ 10^9^ *hr*^− 1^) are obtained for case 1 ~ 3, as shown in Figure 10. The estimated quanta generation rate of the index patient in the outbreak case is 0.141 million, 0.103 million and 0.127 million for case 1 ~ 3, respectively. The likelihood of the no movement case is significantly larger than case 2 and 3 (0.414 comparing with 0.026 and 0.055). So case 1 best fits the infection pattern of the real case among case 1 ~ 3.

**Table 5:** **Grouping of the passengers in the likelihood analysis**

Case	Case 1 (no movement)				Case 2 (0.5 m/s)				Case 3 (1.0 m/s)			
Group	1	2	3	4	1	2	3	4	1	2	3	4
Susceptible people *N*_*s*_	28	24	6	13	28	25	9	9	31	22	6	12
Infected people *n*_*s*_	4	7	2	4	5	7	3	2	8	3	2	4

In case 2 and 3, it was assumed that the moving person would walk through the aisle at a constant speed during each cough of the index patient. However, all of the three movement behaviors (moving speed of 0, 0.5 and 1.0 m/s during each cough) might happen during the flight and the movement of the moving person may not be so frequently. To verify whether a combination of case 1 ~ 3 may be more likely to happen in the real case, a range of combinations of case 1 ~ 3 are also investigated in the likelihood analysis according to Eq. (3):

5 $$\begin{array}{l} D\left(x,t\right)=\left(1-a_{1}-a_{2}\right)D_{1}\left(x,t\right)+a_{1}D_{2}\left(x,t\right)\\ \;+a_{2}D_{3}\left(x,t\right) \end{array}$$

where *D*_1_(*x*,*t*), *D*_2_(*x*,*t*) and *D*_3_(*x*,*t*) are the intake fractions of the susceptible passengers for case 1 ~ 3, respectively, which are calculated by Eq. (2) and shown in Figure 6; *a*_1_ and *a*_2_ are the rates that demonstrate how often the moving person walks in the airplane at moving speeds of 0.5 m/s and 1.0 m/s during the coughs of the index patient, *a*_1_ + *a*_2_ ≤1.

By using likelihood analysis, the likelihood values under a range of quanta generation rate values, *a*_1_ and *a*_2_ can be obtained (data not shown). The result shows that the maximum likelihood is still 0.414 when the quanta generation rate is 0.141 million and both of *a*_1_ and *a*_2_ are zero. So the no movement assumption best represents the outbreak case. This result fits with the findings of a previous study, which analyzed a multi-drug resistant tuberculosis outbreak case in an airplane cabin by using likelihood analysis [18]. Yin et al. [18] did not simulate human movement but using a ‘mixing ratio’ concept to analyze the effect of human movement on the transmission of infectious disease. It was also found in the tuberculosis case that the likelihood reached maximum at mixing ratio close to zero, which indicated that human movement only had insignificant effect on the airborne transmission of respiratory infectious diseases.As shown in Figure 7, the moving person also receives a high intake dose and has a high exposure level. So, if a seated passenger walks in the airplane cabin (e.g. walking to the washroom) when the index patient is coughing, his/her exposure level will also significantly increase and result in a higher infection risk. This effect is also investigated in the likelihood analysis according to Eq. (3):

6 $$\begin{array}{l} D\left(x,t\right)=\left(1-b_{1}-b_{2}\right)D_{1}\left(x,t\right)+b_{1}D_{2}\left(\mathrm{MP},t\right)\\ \;+b_{2}D_{3}\left(\mathrm{MP},t\right) \end{array}$$

where *D*_2_(*MP*,*t*) and *D*_3_(*MP*,*t*) are the intake fractions of the moving person calculated in case 2 and 3, respectively; *b*_1_ and *b*_2_ demonstrate how often the seated passengers walk in the airplane at moving speeds of 0.5 m/s and 1.0 m/s during the coughs of the index patient. So *b*_1_ and *b*_2_ are the average possibilities for all the 71 passengers. Due to the limitation of the space in the aisle and the short influence duration of a cough, it can be assumed that only one person may walk through the aisle during each cough. So the average possibility that each passenger walks in the airplane during a cough of the index patient is 1/71 = 0.014, which means b_1_ + b_2_ ≤0.014.

By using likelihood analysis, the likelihood values under a range of quanta generation rate, *b*_1_ and *b*_2_ can be obtained (data not shown). The maximum likelihood is 0.763, when the quanta generation rate is 1.36 million, *b*_1_ is 0.014 and *b*_2_ is zero. This likelihood value is much higher than that of the previous cases. So, the risk assessment results will better fit the real case when the movement of the seated passengers is considered. An average walking possibility of 1.4% can be used to represent and simulate the movement behaviors of all the seated passengers.

## Discussion

According to the risk assessment results, the average infection risk of all the seventy-one seated passengers increases by 1.7% ~ 2.2% due to human movement. So the frequent walking of the crew members or the passengers will raise the infection risk level in the airplane cabin. However, a 1.7% ~ 2.2% increase is not so significant. By estimating the likelihoods of a series of human movement assumptions, the no movement case has the highest likelihood and is most likely to be the real case. This conclusion fits with the estimated results given by a previous study which stated that no human movement is the most probable case [18]. This result does not imply that there is no human movement in the real outbreak case, which is impossible. The crew members or the passengers may still walk frequently in the airplane when the index patient is not coughing. It needs to be emphasized that although the no movement case is the most likely case in this outbreak case, the conclusion may not be generalized to all outbreak cases because the numerical simulation and the risk assessment are case-dependent.

**Figure 11:**
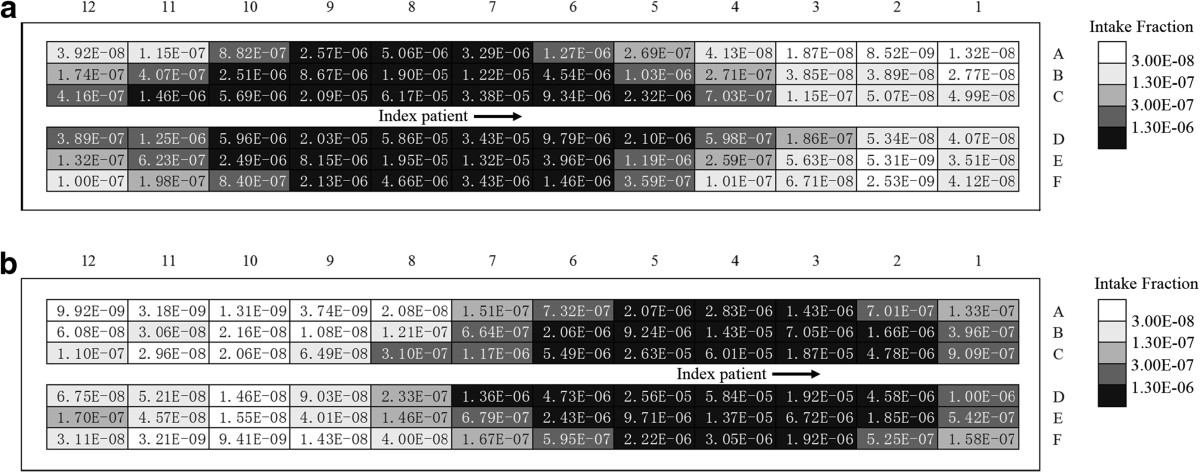
**Intake fraction of each passenger for case 4 and 5. (a)** case 4, **(b)** case 5.

The moving person also has a significantly high intake dose. When walking through the aisle during the coughing of the index patient, the moving person may receive an intake fraction of 2.2 × 10^−6^, which is larger than that of 80% of the seated passengers. Especially, for the passengers who are seated far away from the index patient, the intake fractions that they received during walking in the airplane cabin can be *O*(10^2^) larger than that without movement. If a seated passenger walks in the airplane and passes through the high-dose region close to the index patient, he/she may also receive a significantly high intake dose. Frequent walking of the seated passengers may also result in a high infection risk, which may be up to 55%. After considering the movement behaviors of the seated passengers in the risk assessment and likelihood analysis, a much higher likelihood is estimated. So the walking activities of the seated passengers may be another explanation of the infection of the passengers seated far away from the index patient. This result indicates that the movements of the seated passengers also have a strong influence on the infection risk distribution in the airplane cabin. Taking the influence of human movement into consideration is still necessary and important for the infection control in enclosed environment.The droplets dispersion and disease transmission in the airplane cabin are also highly depended on the behavior characteristics of the index patient, e.g. the index patient coughs during walking along the aisle or standing and waiting outside the lavatory. Figure 11 demonstrates the intake fraction distribution induced by one cough in the airplane cabin for case 4 ~ 5. The index patient was walking (e.g. towards the lavatory) and coughed at different location. Same as the intake fraction distribution shown in Figure 6, local area around the index patient has a significantly higher intake dose than further area. The intake fraction and the infection risk of each passenger is mainly depended on the distance between the passenger and the cough location. Thus, if the index patient coughs during walking in the aisle or waiting outside the lavatory, another high-dose region will be formed around the current location of the index patient, rather than the seat location (9E). The frequent walking of the index patient may influence the infection risk distribution in the airplane cabin and increase the infection risk of the passengers who are seated far from the seat location of the index patient. Passengers who are seated close to the lavatory may also have significantly high infection risks for the index patient may need to use the lavatory frequently. Thus the infection risk distribution in the airplane cabin is also highly depended on the behavior characteristics of the index patient. The risk assessment results will be more accurate when considering the droplets dispersion process under different walking activities of the index patient. So a better understanding and estimation of the behavior characteristics of the index patient will be very important and useful for infection risk assessment and control in airplane cabin.

Uncertainties and errors still exist in this work. In the risk assessment and the likelihood estimation, an average walking possibility is used for all the seated passengers. This is not accurate because each individual may have a unique behavioral characteristic. So investigations on behavioral characteristics of the occupants in the airplane are strongly needed. And also, only the coughing droplets are numerical simulated in this study. Droplets expelled by breathing and sneezing can also influence the infection risk of each individual. An aerosol source model that takes the droplets expelled by multiple respiratory activities into account may also be helpful for better evaluating the aerosol disseminate process in the airplane cabin. The pathogen concentration in the expiratory fluid was considered as constant in this work. Since the volume concentration of the virus varies with the size of the cough droplets, changes of the pathogen concentration in the droplets of different size may also influence the risk assessment results [50]. Besides, the re-suspension of the aerosols is not considered in this work. During human walking, the movement may lead to the re-suspension of the aerosols from the floor. When taking the re-suspension of the exhaled droplets into consideration, the risk assessment results will be more accurate. However, since the airflow induced by the ventilation system is downward to the outlet (located on the side wall and close to the floor), the re-suspended aerosols can hardly rise to the breathing level of the seated passengers. So the effects of the re-suspended aerosols on the risk assessment results can be considered as insignificant in this case.

## Conclusions

In this work, the influence of human movement on airborne disease transmission in an airplane cabin is investigated. An on-flight outbreak case of SARS is chosen to demonstrate the risk assessment process. The Eulerian–Lagrangian approach is used to simulate the dispersion and deposition of infectious droplets expelled by the index patient. The simulation result shows that human movement improves the mixing of the air in the airplane cabin, strengthens the downward transport of the aerosols, and decreases the deposition and removal rate of the aerosols.

The infection risks of the occupants are assessed by using the dose–response model in infection risk assessment. The average infection risk of the seated passengers is 0.2015, 0.2051 and 0.2096 for no human movement, moving speeds of 0.5 m/s and 1.0 m/s, respectively. The assessment result shows that human movement may increase the average infection risk in the cabin, especially for the passengers seated three rows ahead and one row behind the index patient. The likelihood of each case is estimated and the highest likelihood can be found in the no movement case with a quanta generation rate of 0.141 million. So in this SARS outbreak case, the effect of human movement on airborne disease transmission, as it changes the dispersion and mixing pattern of infectious expiratory aerosol, is found to be very insignificant. Since the numerical simulation and the risk assessment are case-dependent, this result may not be generalized to all cases. Moreover, if the seated passengers walk in the airplane cabin, the intake doses of that passengers may also significantly increase and lead to a high infection risks. So the movements of the seated passengers also have a strong influence on the airborne disease transmission in the airplane cabin and may result in significantly higher infection risks for the moving persons.

The results of this work imply that the infection risk distribution in the airplane cabin highly depends on the movement behaviors of the passengers and the index patient. Taking the influence of the movement of the seated passengers into consideration is necessary. A better understanding and estimation of the behavior characteristics of the index patient is also important for infection risk assessment and control in airplane cabin. To reduce the pathogen concentration in the high-dose region close to the index patient, a personal ventilation system may be a feasible solution which is still needed to be carefully designed and verified in airplane cabin operation. Using N95 respirator masks of the index patient or the passengers can also be a simple and viable method to prevent exposure from inhalation the expiratory aerosols. In future studies, investigations on the behaviors characteristics of the passengers during flight will be useful and helpful for infection control.

## Electronic supplementary material

Additional file 1: Supplementary information for the airflow pattern in the airplane cabin and the Nomenclature.(DOC 2 MB)

Below are the links to the authors’ original submitted files for images.

Authors’ original file for figure 1

Authors’ original file for figure 2

Authors’ original file for figure 3

Authors’ original file for figure 4

Authors’ original file for figure 5

Authors’ original file for figure 6

Authors’ original file for figure 7

Authors’ original file for figure 8

Authors’ original file for figure 9

Authors’ original file for figure 10

Authors’ original file for figure 11

Authors’ original file for figure 12
