# Supplementary material for: Effect of human movement on airborne disease transmission in an airplane cabin: study using numerical modeling and quantitative risk analysis
Source: BMC Infect Dis. 2014 Aug 6;14:434. doi: 10.1186/1471-2334-14-434 (PMC4133625; doi:10.1186/1471-2334-14-434)
Supplement: Supplementary file 1 — Additional file 1: Supplementary information for the airflow pattern in the airplane cabin and the Nomenclature.(DOC 2 MB) [file 12879_2013_3736_MOESM1_ESM.doc]

Effect of human movement on airborne disease transmission in an airplane cabin: study using numerical modeling and quantitative risk analysis

Z.Y. Han^1, 2^, G. N. Sze To^3^, S.C. Fu^2^, C. Y. H. Chao^2^, W.G. Weng^1^, Q.Y. Huang^1^

1 Institute of Public Safety Research, Department of Engineering Physics,

Tsinghua University, Beijing 100084, China

2 Department of Mechanical and Aerospace Engineering, The Hong Kong

University of Science and Technology, Clear Water Bay, Hong Kong

^3^ Building Energy Research Center, Fok Ying Tung Graduate School, The Hong Kong University of Science and Technology, Clear Water Bay, Hong Kong

Additional File

**1 Airflow pattern in the airplane cabin**

In this work, the steady-state airflow pattern in the cabin was used as the initial condition for the transient simulation. The velocity vectors of the steady-state airflow pattern are shown in Fig. A1, in the cross-section and longitudinal section through the index patient, respectively. As shown in Fig. A1 (a), the cold air comes into the airplane cabin from the supply inlets, flows downward and sideward through the cabin and exits from the outlets located at the bottom of the sidewalls. From Fig. A1 (b), similar downward airflow can be seen around the passengers. No symmetrical recirculation zone exists in the velocity field shown in Fig. A1, unlike the airflow pattern in the twin-aisle airplane. Fig. A1 also demonstrates that the downward airflow can be found in both sides of the aisle, which is induced by the ventilation system. No up-rising air current can be found due to the significant downward airflow induced by the ventilation system, unlike the upward airflow induced by the human thermal plume. That means in this single-aisle configuration of airplane cabin, the up-rising air current induced by the human thermal plume is not apparent because of the significant downward airflow induced by the ventilation system. The influence area of these downward airflow is also larger than that of the respiratory exhalation flows of the seated passengers, which indicates that the influence of the personal respiratory exhalation flows of the seated passengers on the airflow field is insignificant comparing with that of the airflow induced by the ventilation system.


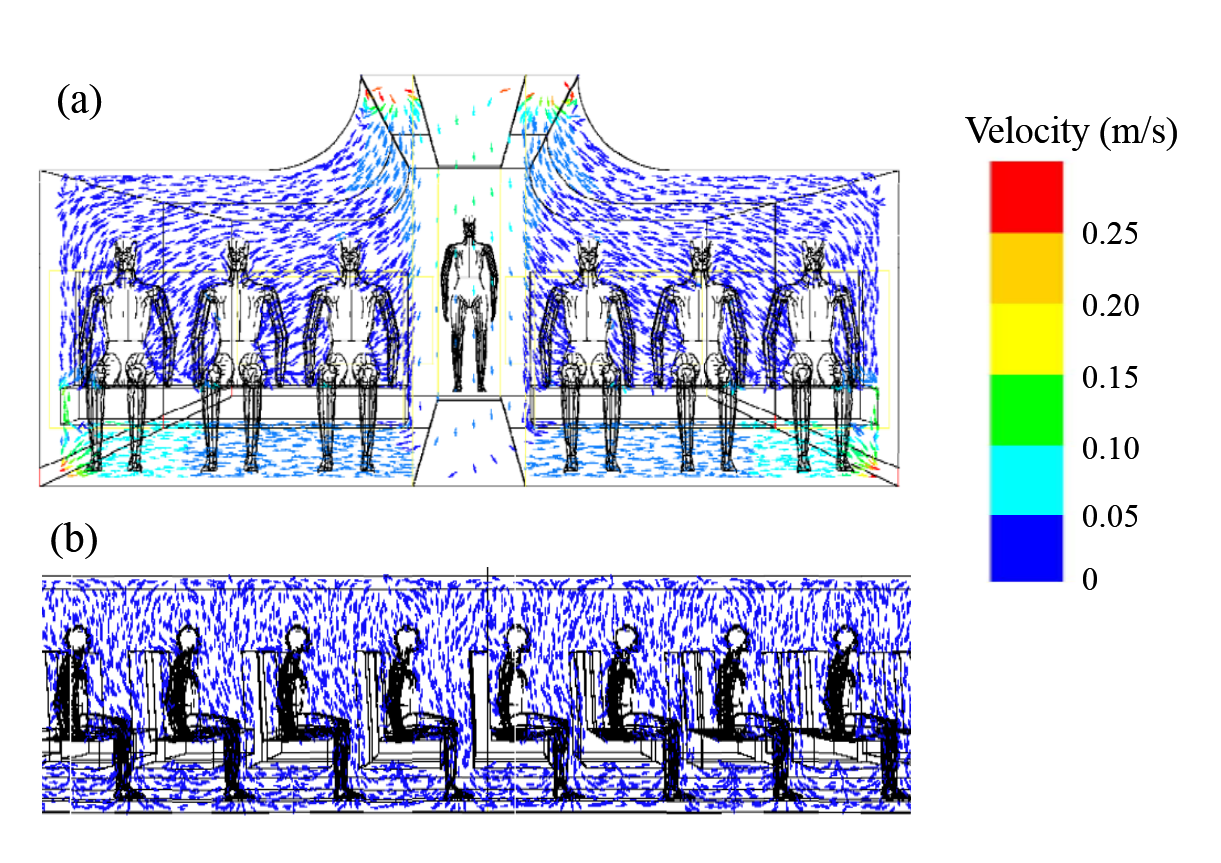


**Fig. A1.** Velocity fields in the airplane cabin of steady- state. (a) cross-section through the index patient, (b) longitudinal section through the index patient.

Although the influence of the airflow induced by the ventilation system on the airflow motion is larger than that of human thermal plume, the heat effects of the seated passengers may still affect the flow field in this airplane cabin. To demonstrate the effects of the heat released from the seated passengers, Fig. A2 shows the temperature distribution in the surrounding area of the seated passengers. It can be seen that the heat released from the human body will significantly change the temperature distribution in the local area around the human body, which may form a up-rising air current when the ventilation is not working.


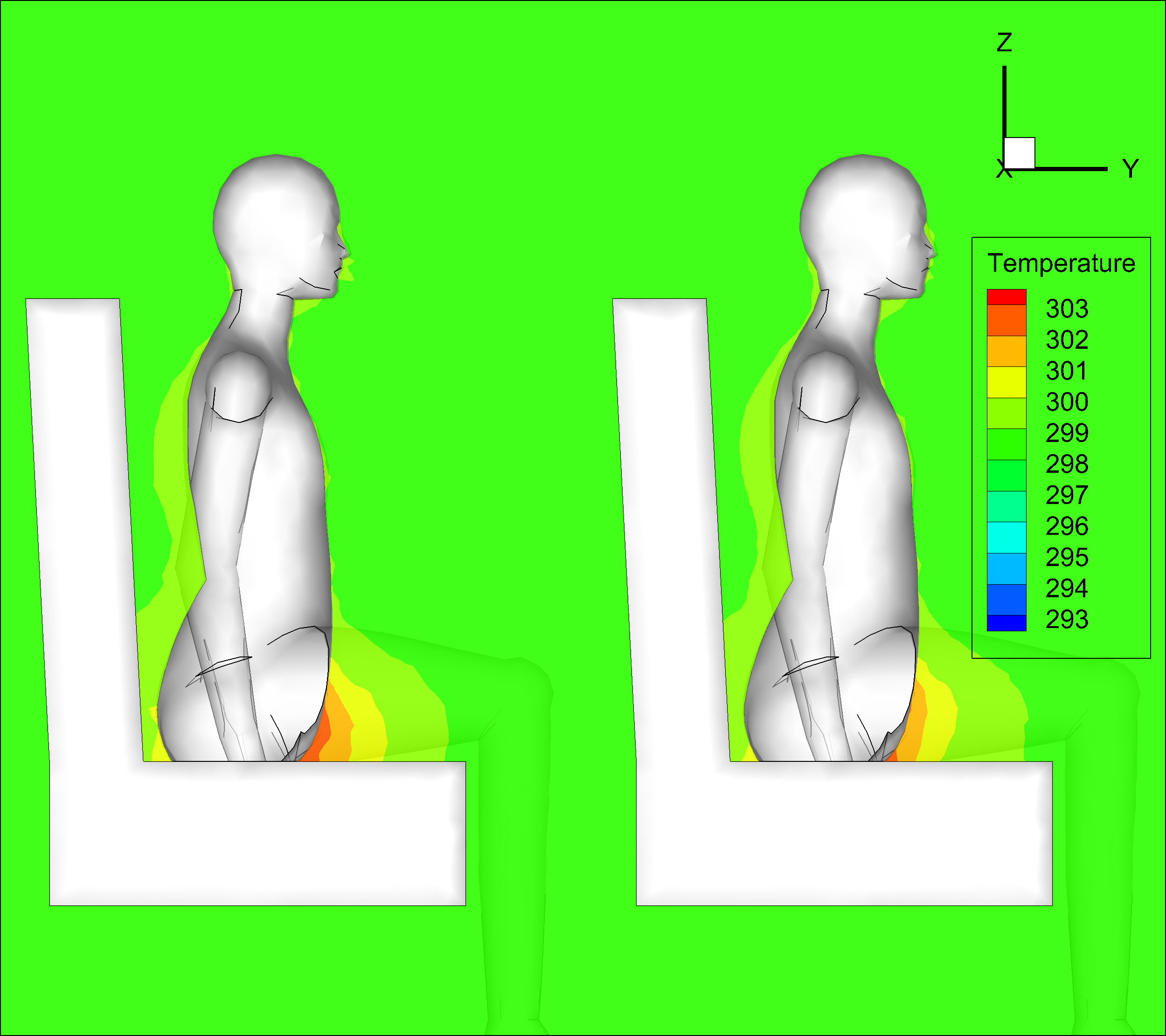


**Fig. A2.** Temperature distribution in cross section around the seated passengers.

When the standing person moves along the aisle, the airflow pattern in the airplane is affected by human movement. Fig. A3 and A4 demonstrate the velocity vectors of the airflow pattern induced by human movement at different times for moving speed of 1m/s, in longitudinal section and horizontal section, respectively. As shown in Fig. A3, when the standing person moves along the aisle, the moving body pushes the air in front of him off the moving path and forms a sideward airflow around the human body. Airflow that follows the movement of the standing person can also be found in the wake behind the human body, similar to the results given by previous studies. Fig. A4 shows that the movement of the standing person pushes the air in front of the human body moving forward. Significant downward airflow exists in the wake behind the torso, which enhances the downward movement of the room air induced by the ventilation system. Fig. A3 and A4 indicate that human movement can disturb the air distribution in a local region and influence the airflow motion in the airplane cabin.


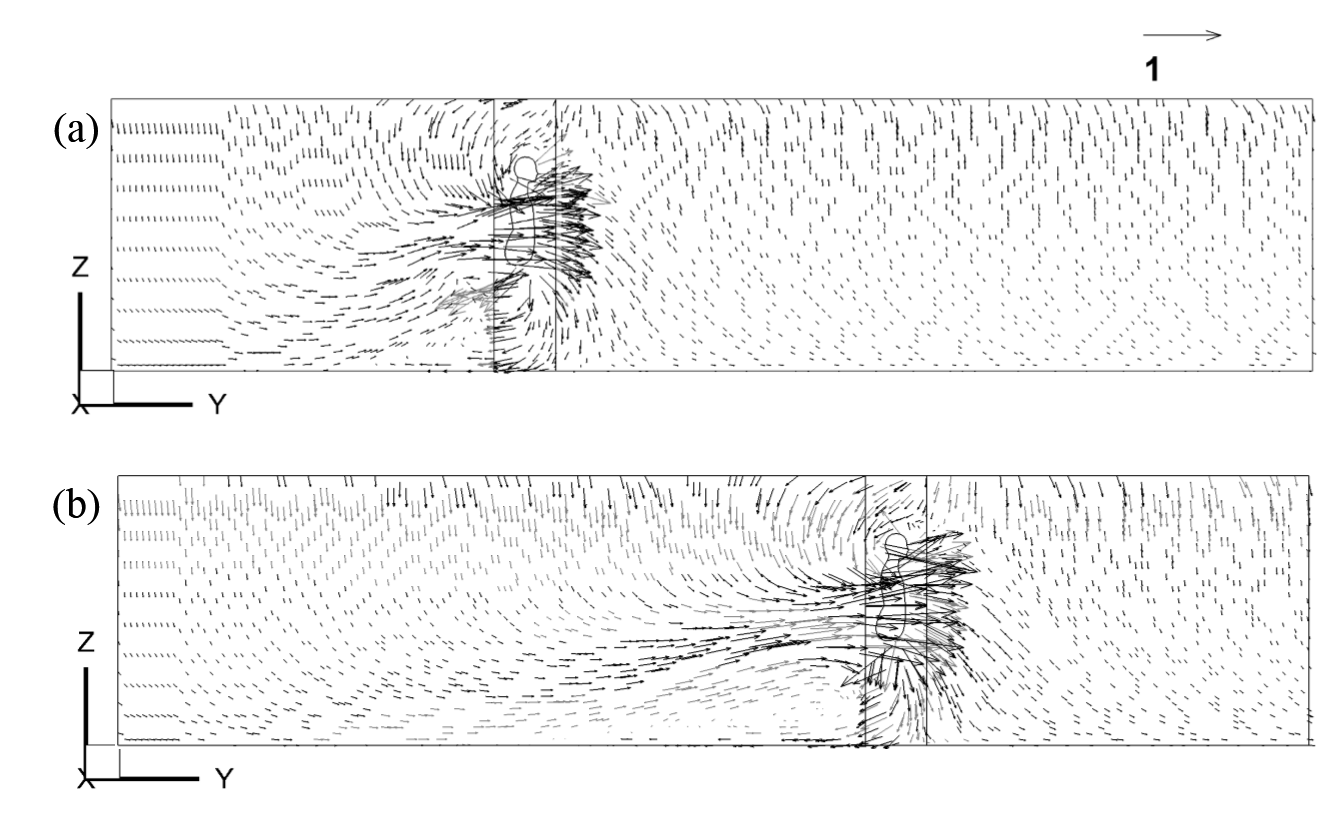


**Fig. A3.** Velocity fields in horizontal section when human moves at 1m/s. (a) t=4s (b) t=7s.


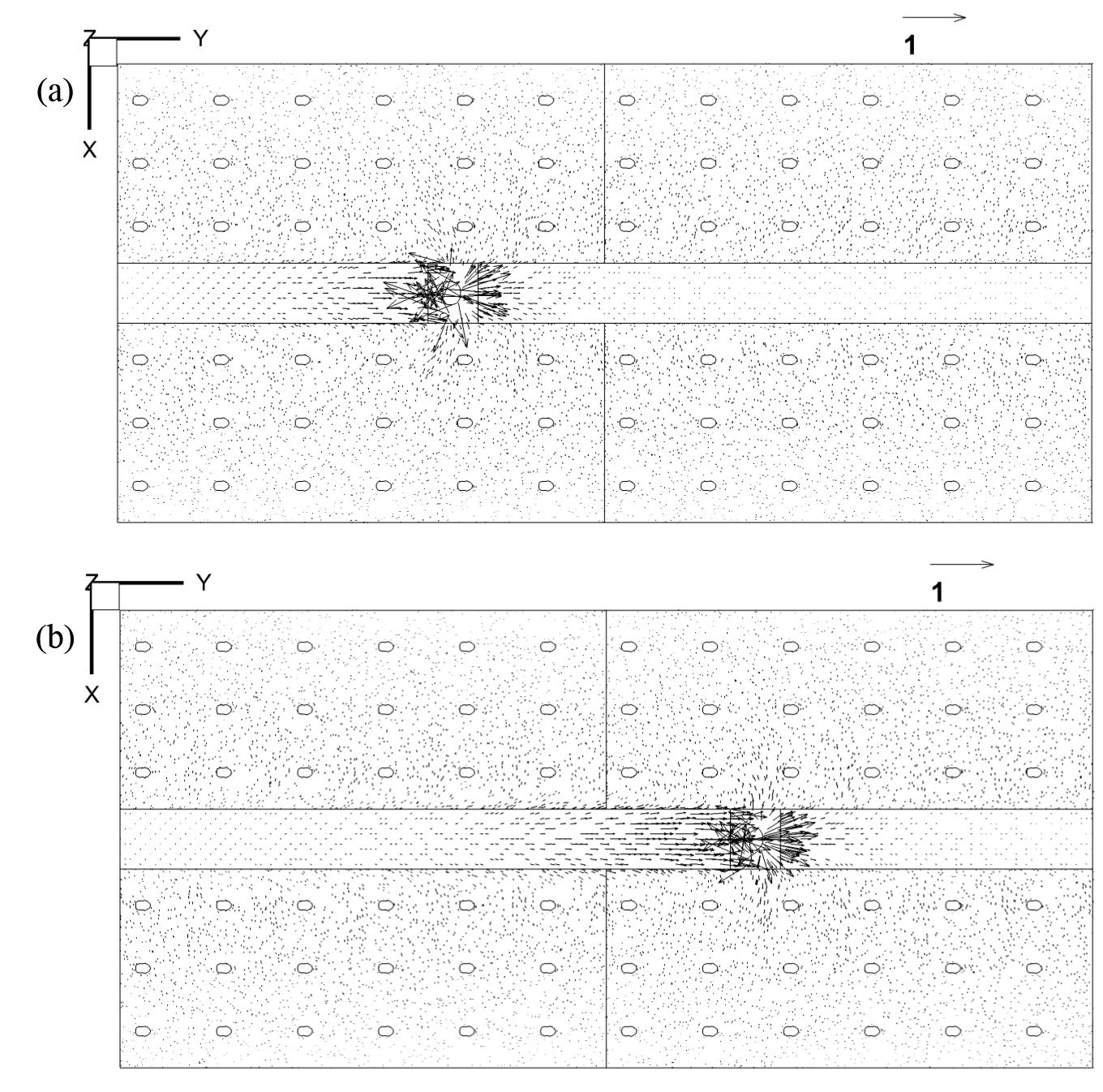


**Fig. A4.** Velocity fields in longitudinal section when human moves at 1m/s. (a) t=4s (b) t=7s.

**2 Nomenclature**

|  | possibility of the movement behavior; |
| --- | --- |
| , | rates that demonstrate how often the moving person walks in the airplane at moving speeds of 0.5m/s and 1.0m/s during the coughs of the index patient, |
| , | rates that demonstrate how often the seated passengers walk in the airplane at moving speeds of 0.5m/s and 1.0m/s during the coughs of the index patient. |
|  | pathogen concentration in the expiratory fluid () |
|  | intake fraction of the susceptible passengers for one cough |
|  | cough frequency () |
|  | viability function of pathogens in the aerosols (%) |
|  | ratio of number of droplets in a cough to the number of injected particles in the numerical model |
|  | average relative likelihood |
|  | total number of size bins |
|  | total quantity of pathogens in aerosols produced in a cough () |
|  | total number of the susceptible people |
|  | total number of infected people |
|  | pulmonary ventilation rate () |
|  | Infection risk (infection possibility) |
|  | infectivity |
|  | total number of the groups of the susceptible people |
|  | time () |
|  | exposure time interval of the flight (hr) |
|  | total volume of droplets produced in a cough () |
|  | volume density of expiratory droplets at the location induced by one cough, of air. |

Greek symbols

|  | deposition fraction of the aerosols in the respiratory tract (%) |
| --- | --- |

Subscript

|  | cartesian coordinate |
| --- | --- |
|  | size bin of the aerosols |
|  | group number of the susceptible people |
|  | number of the movement behavior |
