# Supplementary figures and images for: Effect of human movement on airborne disease transmission in an airplane cabin: study using numerical modeling and quantitative risk analysis
Source: BMC Infect Dis. 2014 Aug 6;14:434. doi: 10.1186/1471-2334-14-434 (PMC4133625; doi:10.1186/1471-2334-14-434)

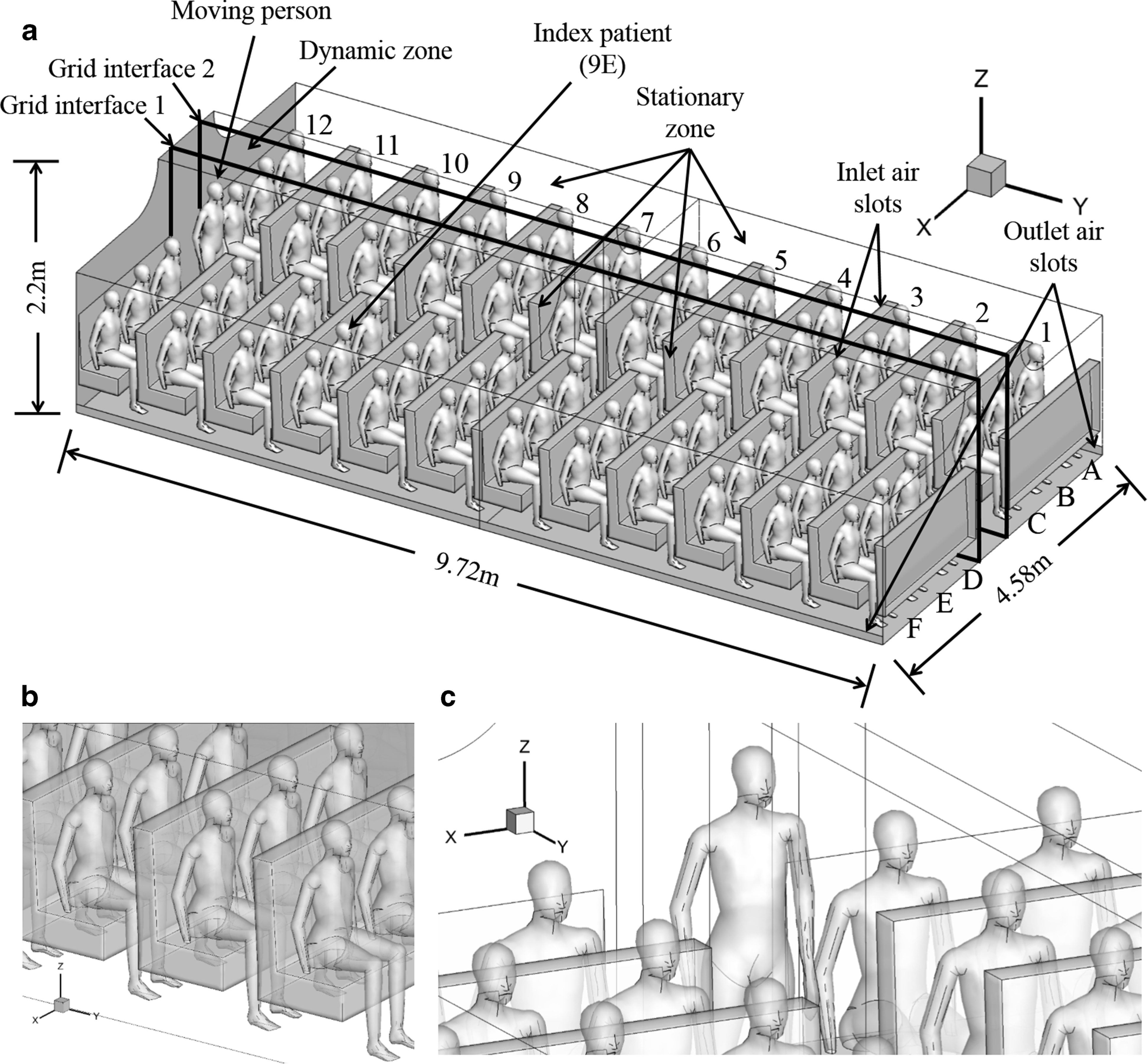

Supplement: Supplementary file 2 — Authors’ original file for figure 1 [file 12879_2013_3736_MOESM2_ESM.tiff]

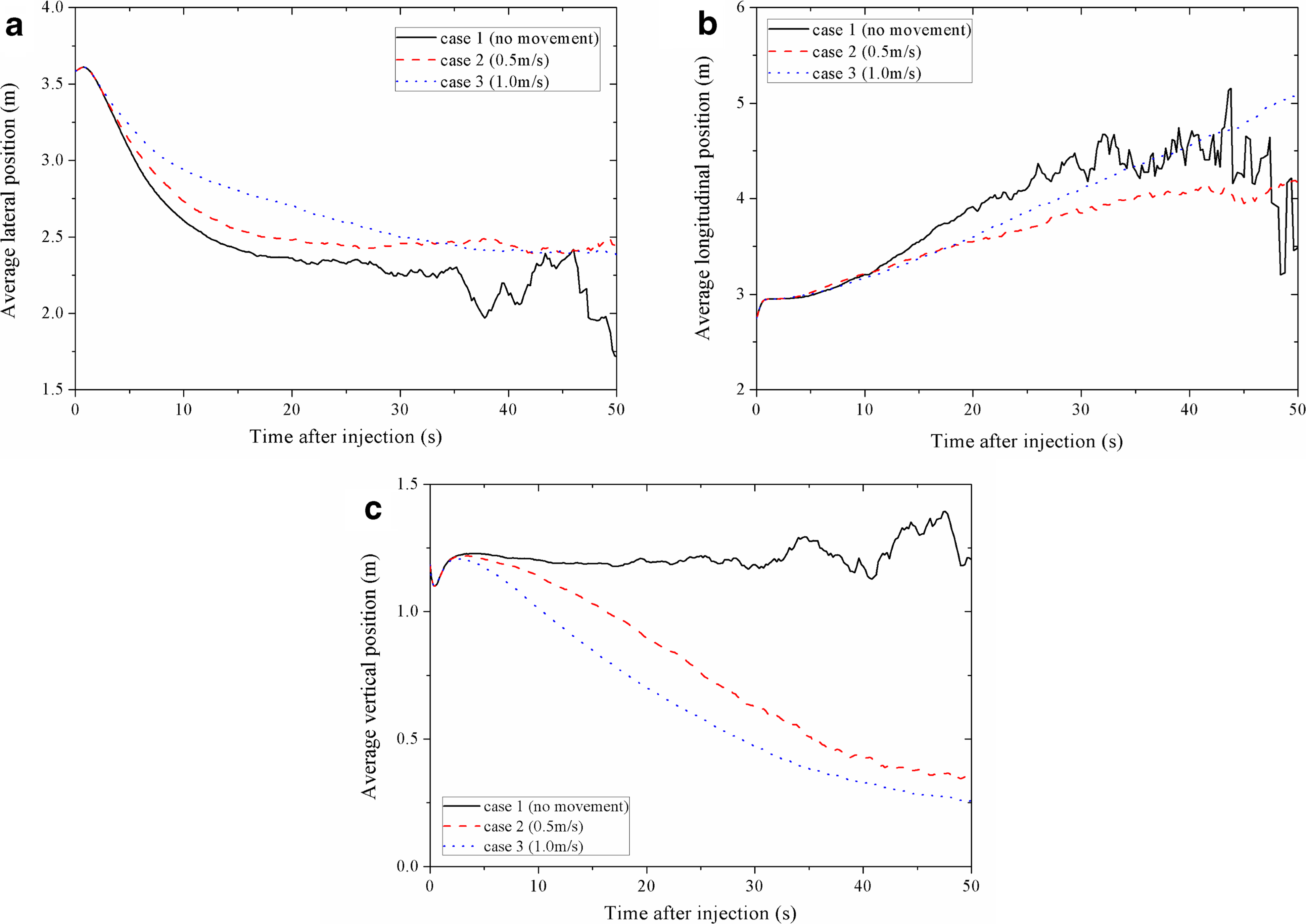

Supplement: Supplementary file 3 — Authors’ original file for figure 2 [file 12879_2013_3736_MOESM3_ESM.tiff]

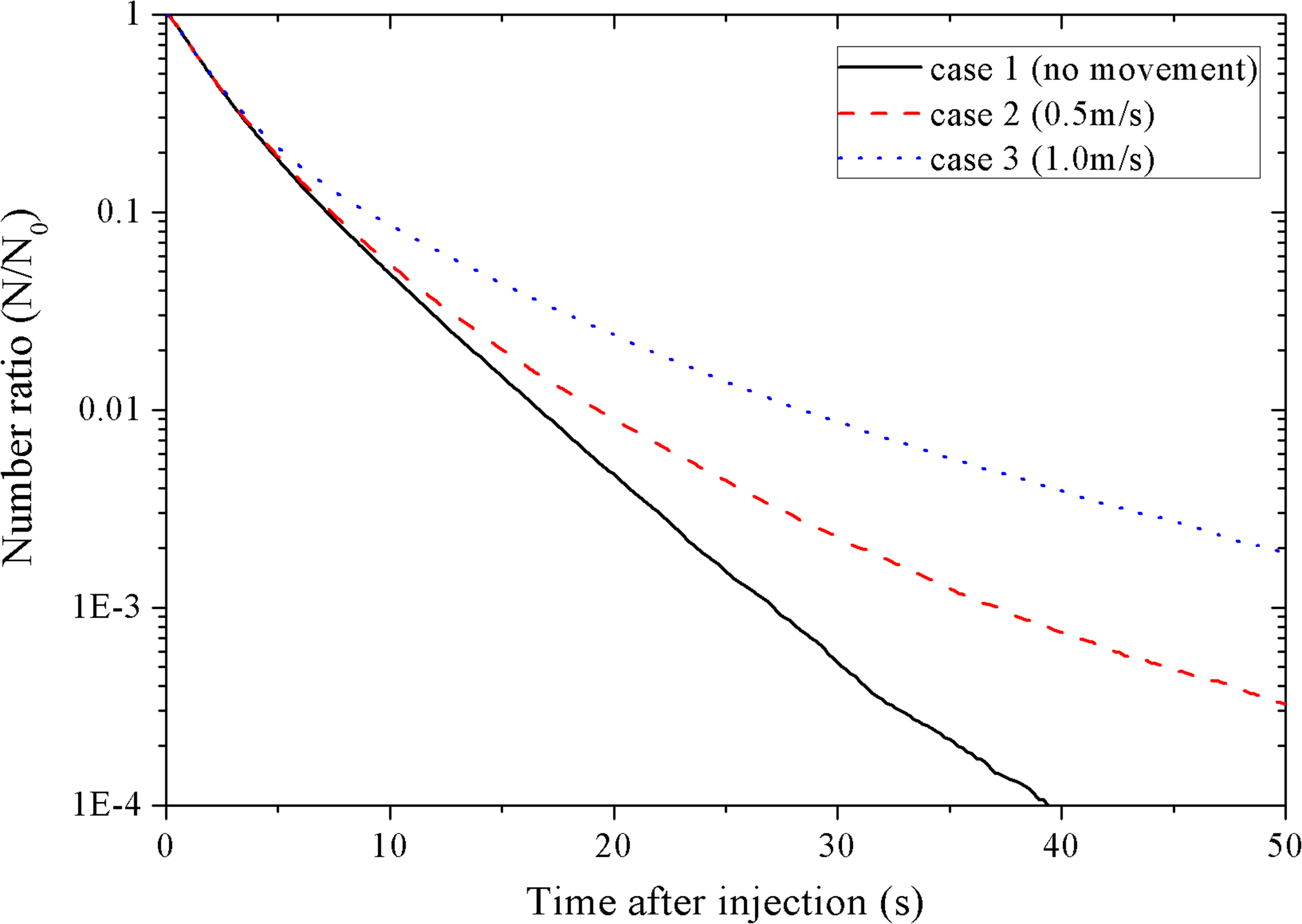

Supplement: Supplementary file 4 — Authors’ original file for figure 3 [file 12879_2013_3736_MOESM4_ESM.tiff]

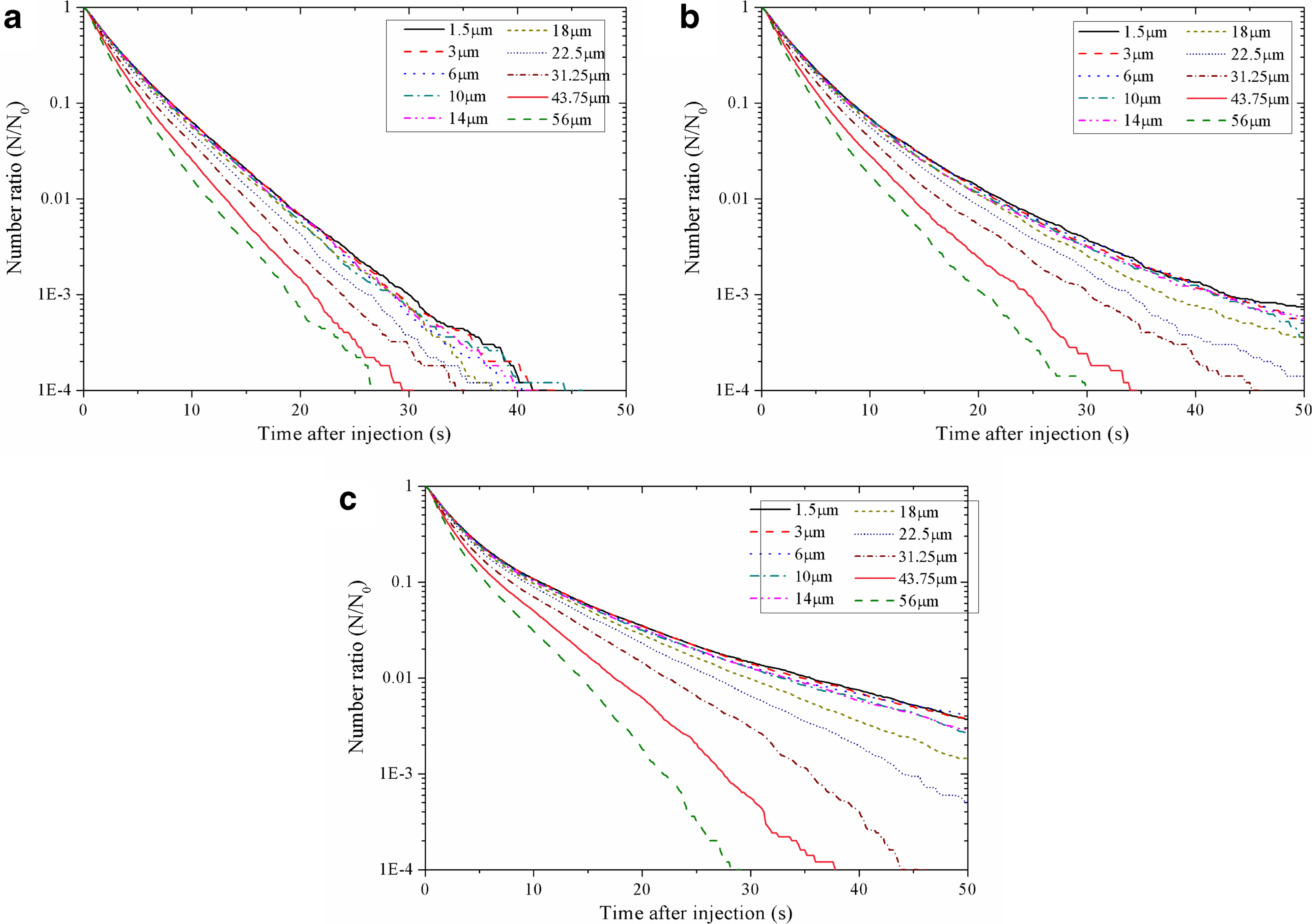

Supplement: Supplementary file 5 — Authors’ original file for figure 4 [file 12879_2013_3736_MOESM5_ESM.tiff]

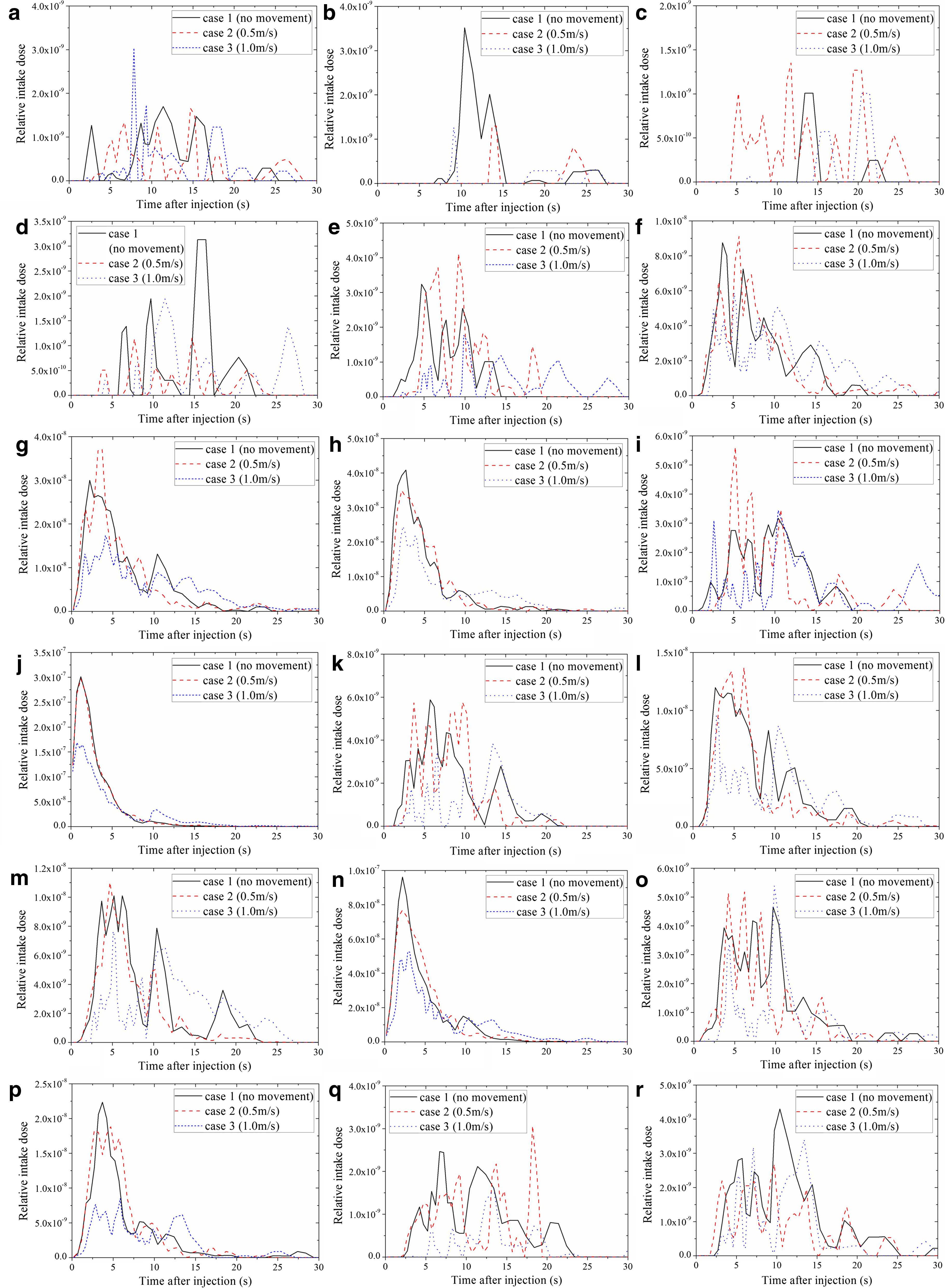

Supplement: Supplementary file 6 — Authors’ original file for figure 5 [file 12879_2013_3736_MOESM6_ESM.tiff]

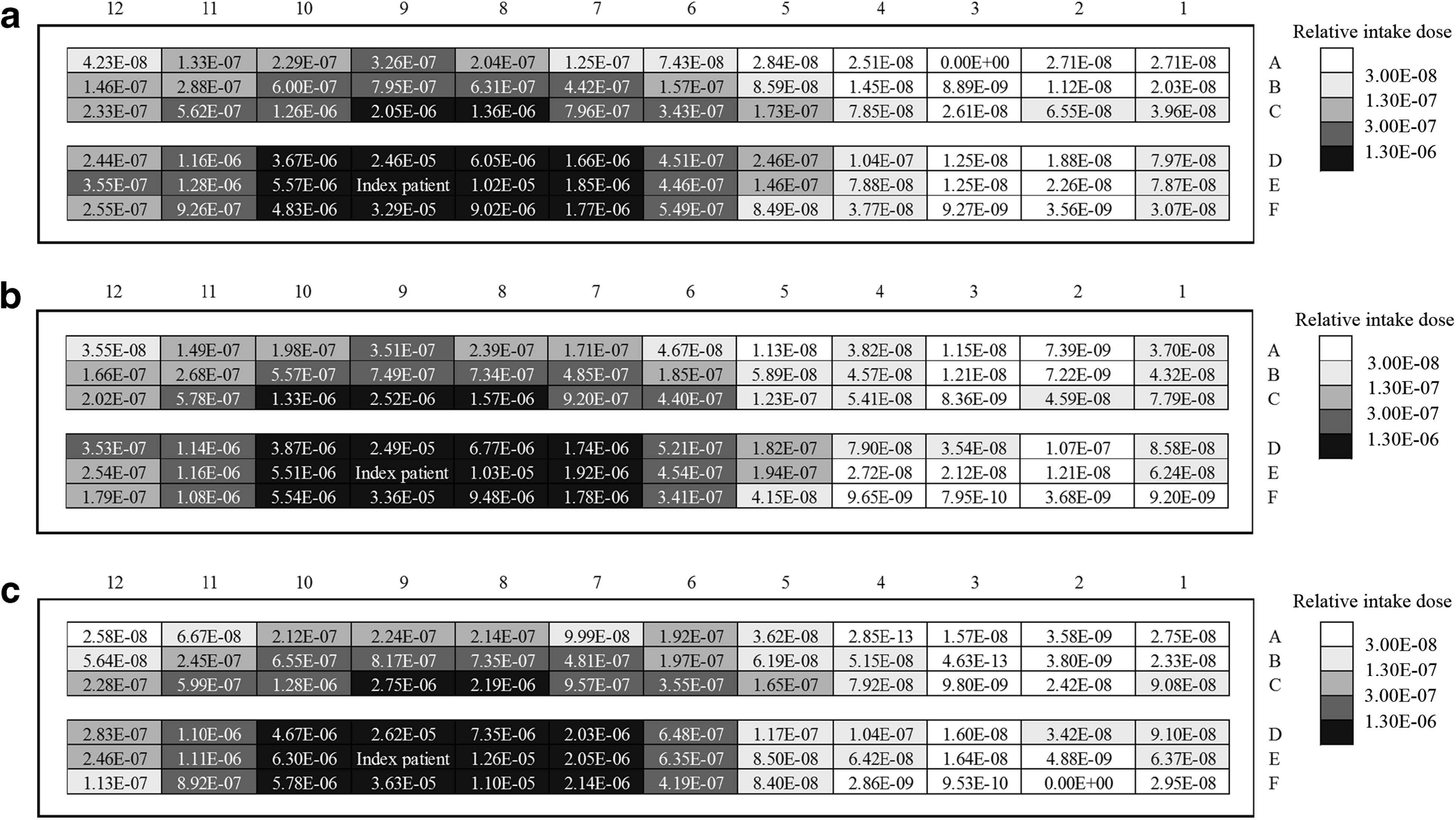

Supplement: Supplementary file 7 — Authors’ original file for figure 6 [file 12879_2013_3736_MOESM7_ESM.tiff]

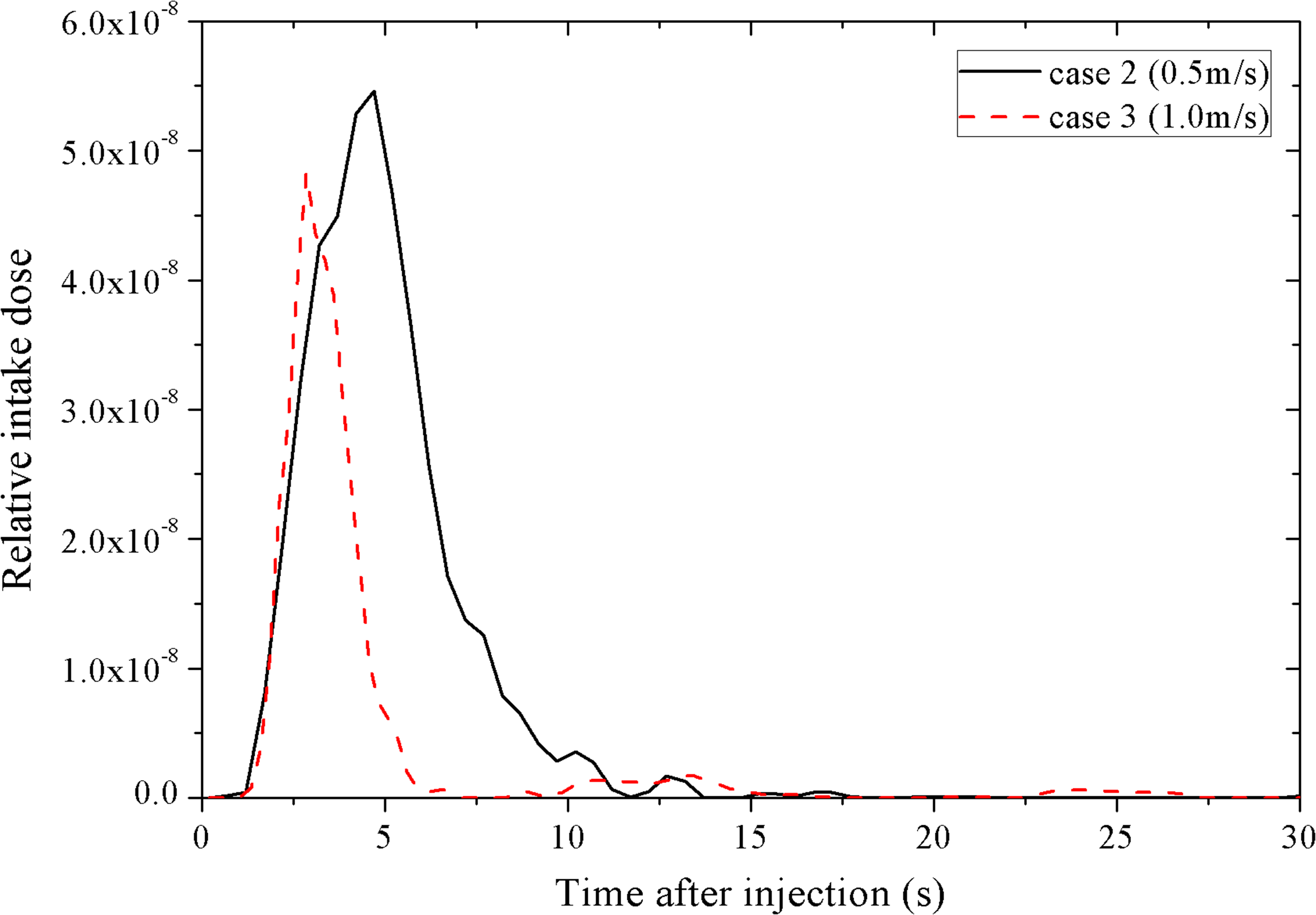

Supplement: Supplementary file 8 — Authors’ original file for figure 7 [file 12879_2013_3736_MOESM8_ESM.tiff]

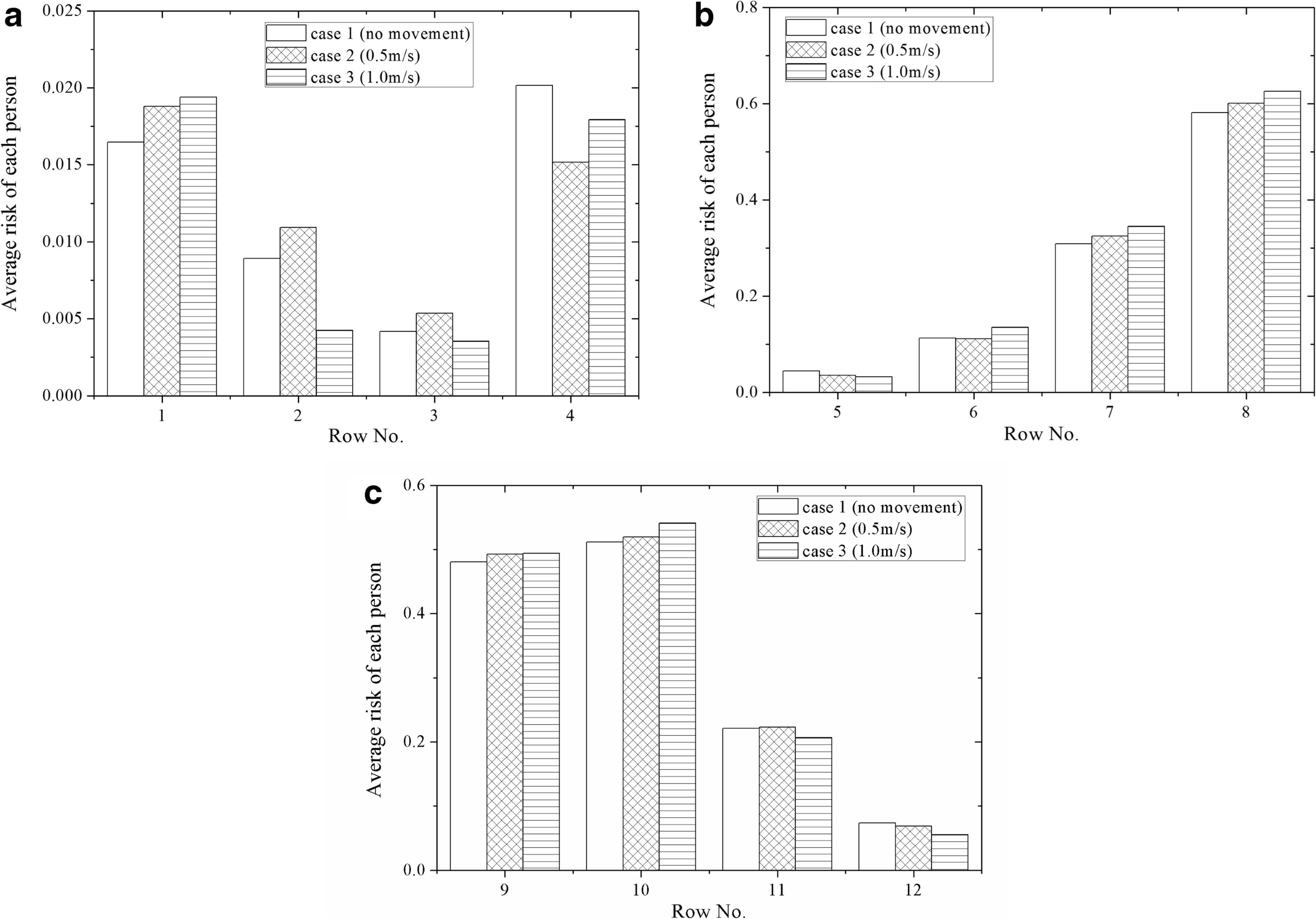

Supplement: Supplementary file 9 — Authors’ original file for figure 8 [file 12879_2013_3736_MOESM9_ESM.tiff]

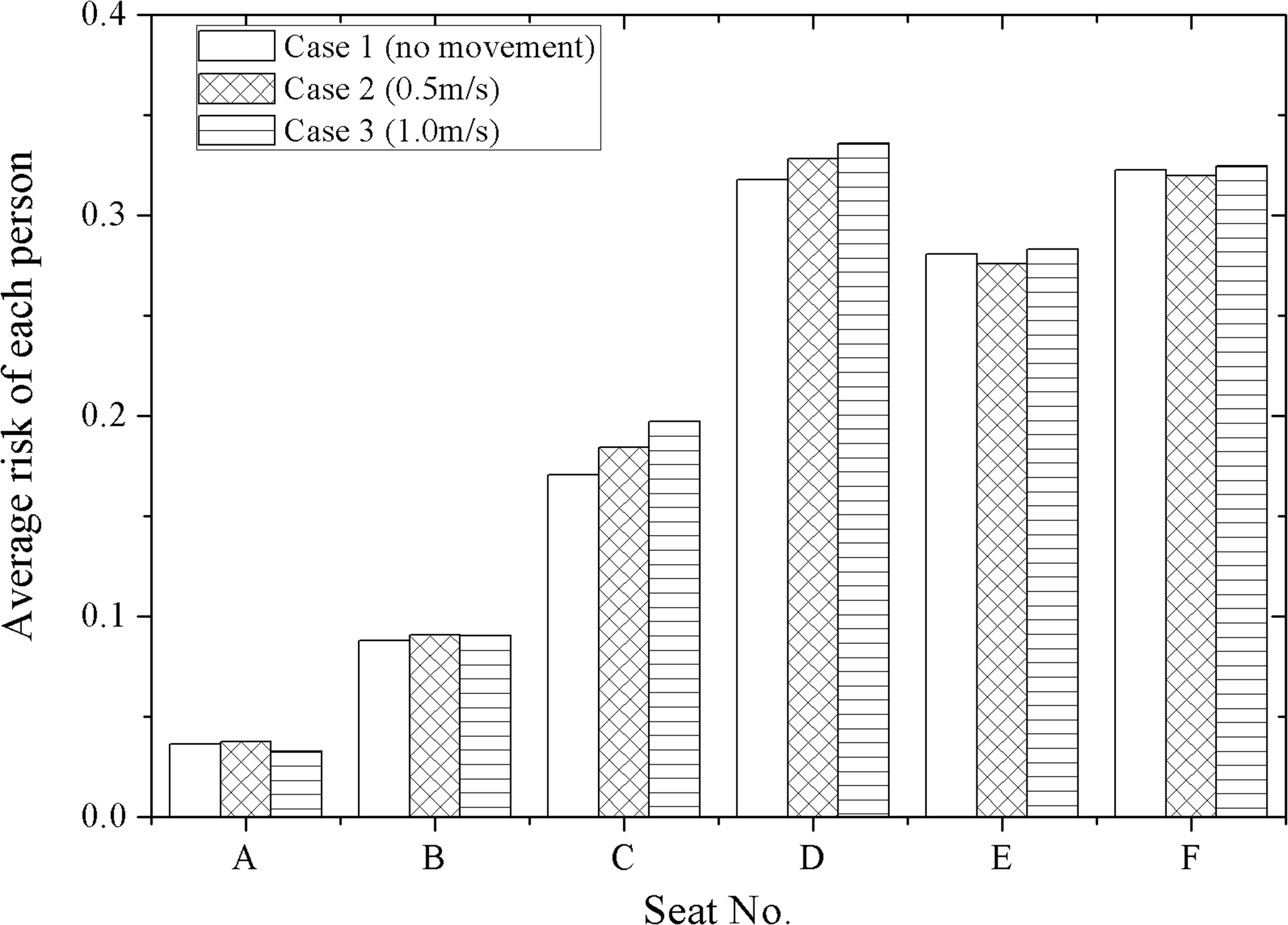

Supplement: Supplementary file 10 — Authors’ original file for figure 9 [file 12879_2013_3736_MOESM10_ESM.tiff]

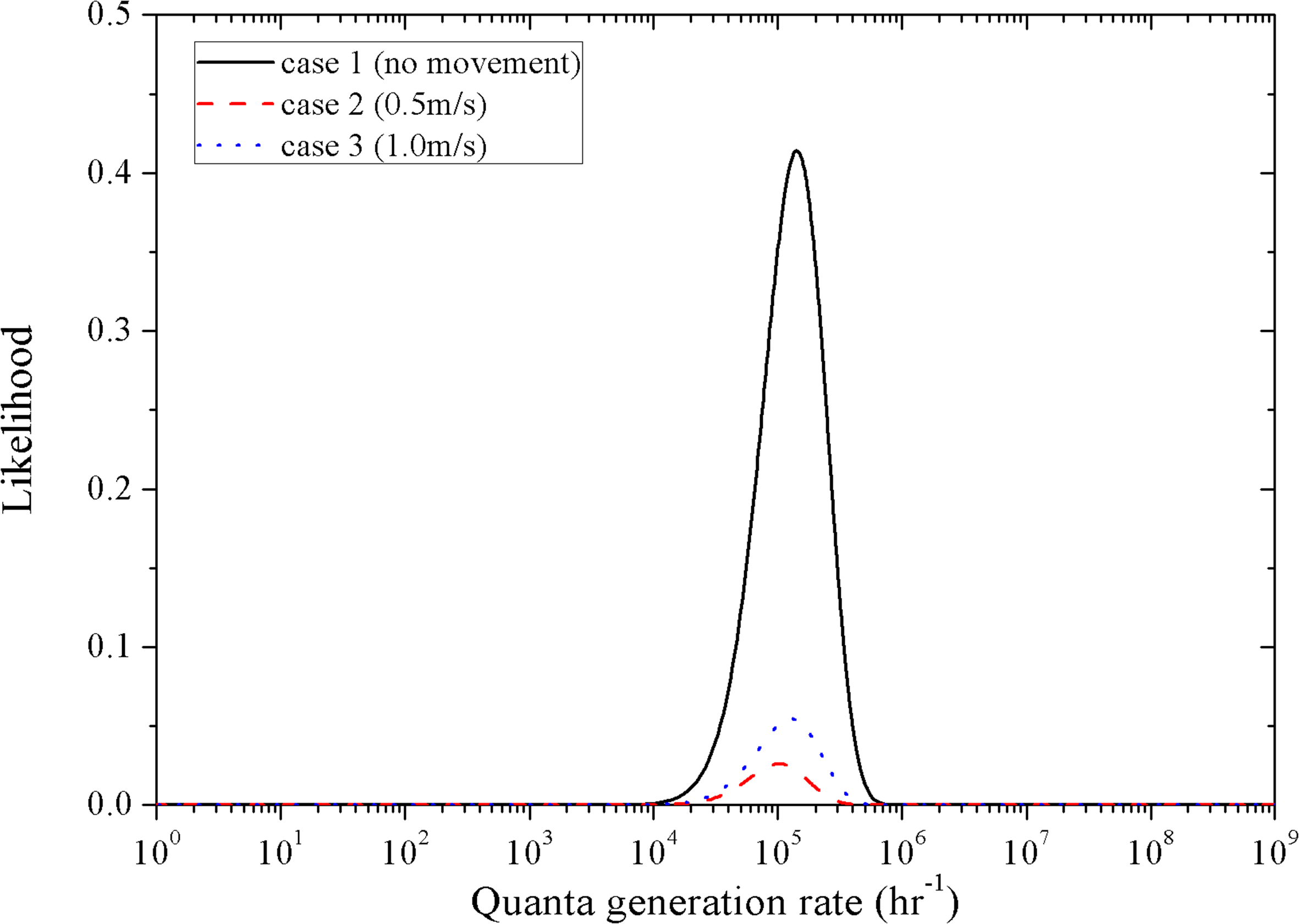

Supplement: Supplementary file 11 — Authors’ original file for figure 10 [file 12879_2013_3736_MOESM11_ESM.tiff]

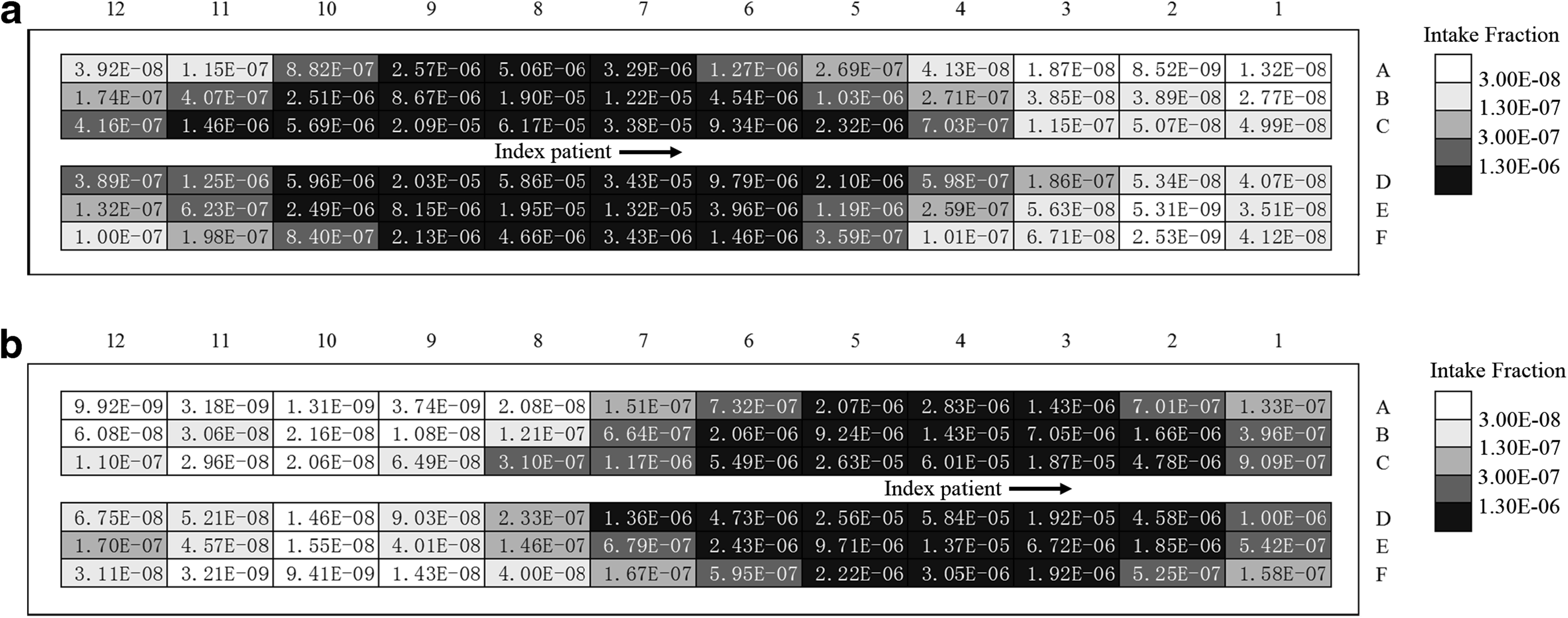

Supplement: Supplementary file 12 — Authors’ original file for figure 11 [file 12879_2013_3736_MOESM12_ESM.tiff]
