# Supplementary material for: Effect of human movement on airborne disease transmission in an airplane cabin: study using numerical modeling and quantitative risk analysis
Source: BMC Infect Dis. 2014 Aug 6;14:434. doi: 10.1186/1471-2334-14-434 (PMC4133625; doi:10.1186/1471-2334-14-434)
Supplement: Supplementary file 13 — Authors’ original file for figure 12 [file 12879_2013_3736_MOESM13_ESM.doc]

**Table 1.** Previous literatures on the effects of human movement on contaminant transmission

| author, date | method | moving object | environment | contaminant | results |
| --- | --- | --- | --- | --- | --- |
| Bjørn and Nielsen [12] | experiment | a life-sized breathing thermal manikin | full-scale test rooms | tracer gas (dinitrogenoxide, N_2_O) | Exhalation and local effects caused by movement may be worth considering if one wishes to contain contaminants in certain areas |
| Matsumoto and Ohba [10] | experiment | a movable heated object | a full-scale room model | \ | The moving object mode and speed showed a significant effect on the air temperature distribution and ventilation effectiveness |
| Shih et al. [13] | CFD | simple object model | an isolated room | tracer gas (carbon dioxide, CO_2_) | The removal of contaminants was not obviously affected by the moving speed |
| Choi and Edwards [15] | CFD | a realistic walking human model | a Room–Room and a Room–Hall configuration | particle | The rate of mass transport increases as the walking speed increases, but the total amount of material transported is more influenced by the initial proximity of the human from the doorway. |
| Mazumdar et al. [14] | CFD | simple object model | a single inpatient ward | tracer gas (sulfur hexafluoride, SF_6_) | The average concentration change in the breathing levels in the ward was generally small |
| Poussou et al. [11] | experiment | a moving object | a one-tenth scale water-based model | dye | Human movement inside enclosed environments could significantly influence contaminant transport and personal exposures to contaminants. |
| Mazumdar et al. [8] | CFD | simple object model | an airplane cabin | dye/ tracer gas | The movement of a crew member or a passenger could carry contaminants in its wake to as many rows as the person passed |
| Wang and Chow [17] | CFD | three different moving human models | an isolation room | expiratory droplets | Human walking disturbed the local velocity field, and the increase of walking speed could effectively reduce the overall number of suspended droplets |
| Choi and Edwards [16] | CFD | a realistic walking human model | a room compartment | tracer gas (sulfur hexafluoride, SF_6_) | Faster walking speed resulted in less mass transport from the contaminated room into the clean room |

**Table 2** The boundary conditions in the numerical simulation.

| Surface | Velocity | Temperature | Humidity ratio | Discrete phase |
| --- | --- | --- | --- | --- |
| Ceiling | No slip | 297K | None | Trap |
| Side wall | No slip | 293K | None | Trap |
| Floor | No slip | 296K | None | Trap |
| Human body | No slip | 305K | None | Trap |
| Supply air | 2.994m/s  (9.7L/s per person ) | 294K | RH 20% (0.004895) | Reflect |
| Seat | No slip | adiabatic | None | Trap |
| Outlet | Outflow | | | Escape |
| Nose and mouth of the index patient | 10m/s, t=0-0.1s  6m/s, t=0.1-0.2s  4m/s, t=0.2-0.3s  2m/s, t=0.3-0.4s  0m/s, t>0.4s  Open area :0.000968m^2^ | 310.15K | RH 50% (0.01224) | t=0-0.4s, Reflect  t>0.4s, Trap |
| Back and front surface | Periodic | | | Escape |

**Table 3** Size distribution of the aerosol injections.

| Diameter (μm) | Original size (μm) | Total number | Size distribution [34] |
| --- | --- | --- | --- |
| 1.5 | 3 | 50000 | 0.0105 |
| 3 | 6 | 50000 | 0.0610 |
| 6 | 12 | 50000 | 0.2040 |
| 10 | 20 | 50000 | 0.3365 |
| 14 | 28 | 50000 | 0.1830 |
| 18 | 36 | 50000 | 0.0883 |
| 22.5 | 45 | 50000 | 0.0463 |
| 31.25 | 62.5 | 50000 | 0.0231 |
| 43.75 | 87.5 | 50000 | 0.0294 |
| 56 | 112 | 50000 | 0.0179 |

**Table 4** The cases investigated in the numerical simulation.

| Case No. | Injection person | Injection time | Movement start time (s) | Moving speed (m/s) |
| --- | --- | --- | --- | --- |
| 1 | Index person at 9E | t=0s | \ | 0 |
| 2 | Index person at 9E | t=0s | t=1s | 0.5 |
| 3 | Index person at 9E | t=0s | t=1s | 1.0 |
| 4 | Moving person | t=3s | t=0s | 0.5 |
| 5 | Moving person | t=6s | t=0s | 0.5 |

**Table 5** Grouping of the passengers in the likelihood analysis.

| Case | Case 1 (no movement) | | | | Case 2 (0.5m/s) | | | | Case 3 (1.0m/s) | | | |
| --- | --- | --- | --- | --- | --- | --- | --- | --- | --- | --- | --- | --- |
| Group | 1 | 2 | 3 | 4 | 1 | 2 | 3 | 4 | 1 | 2 | 3 | 4 |
| Susceptible people | 28 | 24 | 6 | 13 | 28 | 25 | 9 | 9 | 31 | 22 | 6 | 12 |
| Infected people | 4 | 7 | 2 | 4 | 5 | 7 | 3 | 2 | 8 | 3 | 2 | 4 |
